# Mitochondria protect against an intracellular pathogen by restricting access to folate

**DOI:** 10.1126/science.adr6326

**Published:** 2025-08-14

**Authors:** Tânia Catarina Medeiros, Jana Ovciarikova, Xianhe Li, Patrick Krueger, Tim Bartsch, Silvia Reato, John C. Crow, Michelle Tellez Sutterlin, Bruna Martins Garcia, Irina Rais, Kira Allmeroth, Matías D. Hartman, Martin S. Denzel, Martin Purrio, Andrea Mesaros, Kit-Yi Leung, Nicholas D.E. Greene, Lilach Sheiner, Patrick Giavalisco, Lena Pernas

**Affiliations:** 1 Metabolism of Infection Group, Max Planck Institute for Biology of Ageing, Cologne, Germany; 2 Glasgow Centre for Parasitology, University of Glasgow, Scotland, UK; 3 Dept. Microbiology, Immunology & Molecular Genetics, University of California Los Angeles, USA; 4 Howard Hughes Medical Institute, Chevy Chase, MD USA; 5 Metabolic and Genetic Regulation of Ageing, Max Planck Institute for Biology of Ageing, Cologne, Germany; 6 Phenotyping Core Facility, Max Planck Institute for Biology of Ageing, Cologne, Germany; 7 Developmental Biology & Cancer Department, UCL, Great Ormond Street Institute of Child Health, University College London, UK; 8 Metabolomics Core Facility, Max Planck Institute for Biology of Ageing, Cologne, Germany

## Abstract

As major consumers of cellular metabolites, mitochondria are poised to compete with invading microbes for the nutrients they need to grow. Whether cells exploit mitochondrial metabolism to protect from infection is unclear. Here, we found that the transcription factor ATF4 activates a mitochondrial defense based on the essential B vitamin folate. During infection of cultured mammalian cells with the intracellular pathogen *Toxoplasma gondii*, ATF4 increased mitochondrial DNA levels by driving the one-carbon metabolism processes that use folate in mitochondria. Triggered by host detection of mitochondrial stress induced by parasite effectors, ATF4 limited *Toxoplasma* access to folates required for dTMP synthesis, thereby restricting parasite growth. Thus, ATF4 rewires mitochondrial metabolism to mount a folate-based metabolic defense against *Toxoplasma*.

A mammalian cell can contain hundreds to up to thousands of mitochondria (1). In a healthy cell, mitochondria are viewed as powerhouses that produce ATP and anabolic precursors, and signaling hubs that regulate diverse cellular programs (2). To carry out these functions, these organelles consume large amounts of cellular nutrients (3). In an infected cell however, mitochondria are perceived as targets for intracellular microbes. However, mitochondria can function as nutrient competitors during infection, thereby depriving intracellular pathogens access to key nutrients required for growth (4, 5).

To investigate the cellular programs that rewire mitochondrial metabolism during infection, we focused on mitochondrial DNA (mtDNA). Unlike other organelles, mitochondria have their own genome, a vestige of their prokaryotic origin (6). To replicate their genome, mitochondria depend on cytosolic resources such as those sustained by the B vitamin folate (7). This essential cofactor is required for a broad set of reactions known as one-carbon (1C) metabolism that occur in the cytosol and mitochondria, and are crucial for biochemical processes including the synthesis of purines and thymidine monophosphate (dTMP), both of which are used to generate deoxynucleotide triphosphates (dNTPs) for DNA replication (7). Thus, mtDNA levels reflect mitochondrial use of various cellular resources and dysregulated 1C metabolism and aberrant dNTP pools are linked to mtDNA replication defects (8–10).

Several intracellular pathogens also require host products downstream of 1C metabolism for their growth. Host cells have thus evolved defense mechanisms to limit 1C metabolite availability, such as through the degradation of dNTPs during viral infection (5, 11). However, whether cells actively reprogram mitochondria to restrict pathogen access to 1C metabolism-linked nutrients is unknown. We used the intracellular human parasite *Toxoplasma gondii,* which salvages host resources that depend on 1C metabolism, to investigate the dynamics of mitochondrial metabolism during infection (12, 13).

## ATF4 regulates mtDNA levels during infection and infection-independent stress

**Fig. 1.**
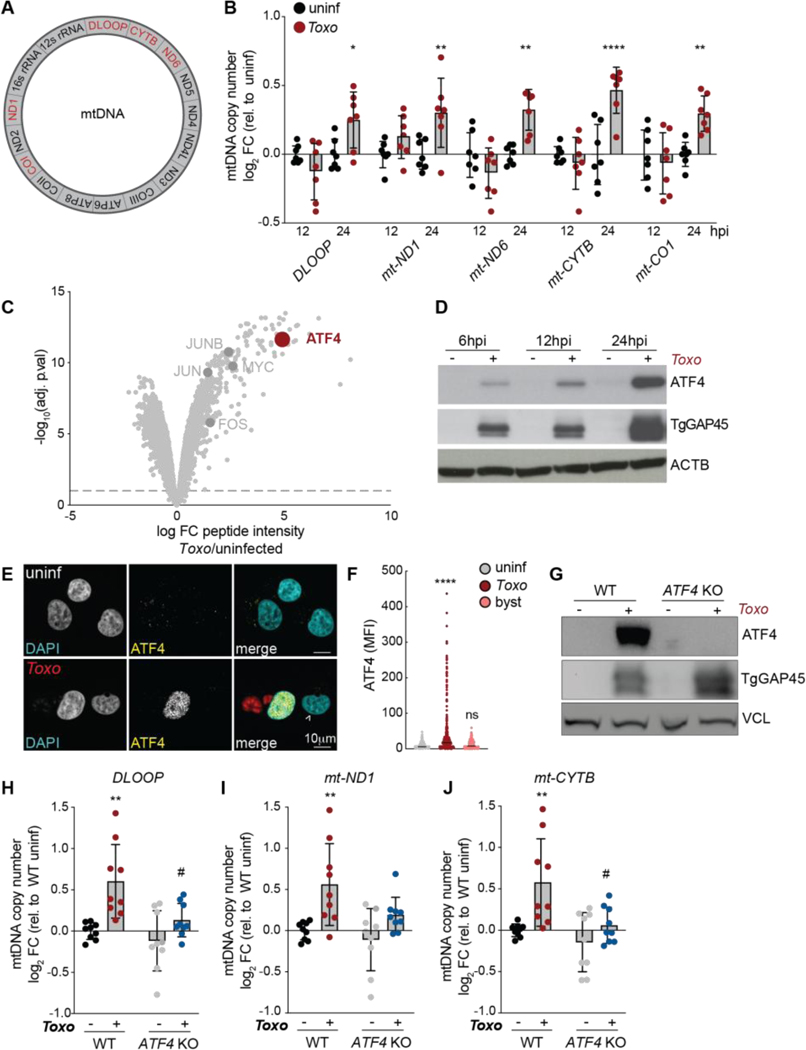
ATF4 increases mtDNA levels during *Toxoplasma* infection. (**A**) Schematic of human mitochondrial genome, genes analyzed by qPCR in red. (**B**) mtDNA levels monitored by qPCR (normalized to *RUNX2*) in ES-2s cells that were uninfected (uninf) or infected with *Toxoplasma* (*Toxo*) at a multiplicity of infection (MOI) of 4 at 12 and 24 hours post infection (hpi). Data are mean ± SD of n=7 independent cultures; *p<0.05; **p<0.01; ****p<0.0001 for uninfected versus infected by means of two-way ANOVA analysis. (**C**) Volcano plot of whole cell lysates from uninfected and infected HeLa cells at 24 hpi and analyzed by means of mass spectrometry with highlighted transcription factors. (**D**) ES-2 cells were uninfected or *Toxo-*infected (MOI: 4) for 6, 12, and 24 hours and analyzed by means of immunoblotting for ATF4, ~50 kDa; β-Actin (ACTB), ~45 kDa; and *Toxoplasma* GAP45 (TgGAP45), ~45 kDa. (**E**) Immunofluorescence images of uninf cells and cells infected with mCherry-expressing *Toxoplasma* at 24 hpi. Arrowhead indicates an uninfected bystander cell that is proximal to an infected cell; scale bar, 10 μm. (**F**) Quantification of mean fluorescence intensity (MFI) of ATF4 in the nuclei of cells in images as in (E); byst: bystander cell. ****p<0.001 for comparison to uninf by means of one-way ANOVA analysis (**G**) Immunoblot analyses of lysates from uninfected and *Toxoplasma-*infected (MOI: 4) WT and *ATF4* KO ES-2 cells at 24hpi: ATF4, ~50 kDa; Vinculin (VCL), ~124 kDa; and *Toxoplasma* GAP45 (TgGap45), ~45 kDa. (**H, I** and **J**) mtDNA levels monitored by qPCR of *DLOOP*, mt-*ND1* and mt-*CYTB* (normalized to *RUNX2*) in uninfected and *Toxoplasma-*infected (MOI: 4) ES-2 cells at 12 at 24 hpi. Data are mean ± SD of n=9 independent cultures; **p<0.01, ***p<0.001 for uninfected versus infected; #p<0.05; ##p<0.01 for WT vs. *ATF4* KO by means of two-way ANOVA analysis. (**B**-**J**) ES-2 cells used for all experiments.

To determine whether infection leads to changes in host mtDNA copy number, we measured mtDNA levels in human ES-2 ovarian cancer cells infected with Type I *Toxoplasma* parasites. We examined levels of *DLOOP*, the regulatory region of mtDNA, and 4 genes at different locations on the mitochondrial genome: *mt-ND1*, *mt-ND6*, *mt-CYTB*, and *mt-CO1* (Fig. 1A). At 24 hours post infection (hpi), the copy number of each gene was increased by ~30% (Fig. 1B). Similar results were obtained in HeLa cells and human fibroblasts (HFF) (Fig. S1). To test whether *Toxoplasma* infection increased mtDNA synthesis, we incubated uninfected and infected cells with the thymidine analog 5-ethynyl-2-deoxyuridine (EdU) that is incorporated into newly synthesized DNA, and compared the abundance of EdU foci per mtDNA nucleoid. Infection increased the number of EdU foci per mtDNA foci (Fig. S2A-B). However, we did not detect cytosolic mtDNA nor the consequent activation of innate immune genes including ISG15 and IRF7 (Fig. S2C-D) (14, 15). Thus, *Toxoplasma* infection drives an increase in intramitochondrial DNA levels.

We noted that infection increased the expression of promoters of mitochondrial biogenesis including nuclear factor erythroid 2-related factor 2 (NRF2) and peroxisome proliferator-activated receptor-γ coactivator 1a (PGC1a) (Fig. S3A-B) (16). To determine whether the increase in mtDNA was due to infection-induced mitochondrial biogenesis, we compared nuclear and mitochondrially encoded transcripts’ levels including *SDHB* and mt-ND1 in uninfected and *Toxoplasma-*infected cells. Infection did not increase their levels (Fig. S3C-D). Consistent with these results, flow cytometry-based analysis of mitochondrial mass revealed minimal differences between uninfected and infected cells at 6, 12, and 24 hpi (Fig. S3E). Furthermore, the expression of enzymes involved in host novo nucleotide synthesis and cytoplasmic nucleotide salvage were mostly unchanged (Fig. S3F-G). Thus, mtDNA copy number is increased during infection independently of global changes in mitochondrial abundance.

How do *Toxoplasma-*infected cells increase mtDNA levels? We noted that infection also induced the expression of the mtDNA transcription factor *TFAM* and mtDNA helicase *TWNK,* whose mild overexpression increases mtDNA levels by ~1.5x-fold, comparable to changes we observed during infection (Fig. S4, Fig. 1B) (17, 18). We posited that the increase in mtDNA was regulated by an infection-induced transcription factor upstream of *TFAM* and *TWNK*. To address this possibility, we compared the protein abundance in whole cell extracts from uninfected cells and *Toxoplasma-*infected cells using mass spectrometry (Table S1). Infection with *Toxoplasma* caused an increase in the abundance of several transcription factors including c-MYC, JUN, and FOS, in line with previous observations (Fig. 1C) (19–21). However, the activating transcription factor 4 (ATF4) was the highest induced host transcription factor with an ~16x-fold enrichment in proteomes of infected cells (Fig. 1C).

ATF4 is the main effector of the integrated stress response (ISR), which promotes cellular recovery during various stresses including ER stress and viral infection (22). Recently ATF4 has been recognized as a key regulator of mitochondrial stress responses (23). Furthermore, the ISR promotes mtDNA recovery in cells with mtDNA double-stranded breaks (24). Indeed, the treatment of cells with tunicamycin, an inhibitor of N-linked glycan biosynthesis that activates the ISR and thus ATF4, was sufficient to increase mtDNA levels independently of infection (Fig. S5). We thus sought to address the role of ATF4 in mtDNA dynamics during infection. To first confirm our proteomics results, we analyzed uninfected cells and *Toxoplasma-*infected cells by immunoblot analysis. Infection induced ATF4 as early as 6 hpi and maximally at 24 hpi, and ATF4 nuclear translocation was only evident in infected cells (Fig. 1D-F). Similar results were obtained in cells infected with the less virulent Type II strain of *Toxoplasma,* indicating that ATF4 activation is a general consequence of *Toxoplasma* infection (Fig. S6A-C). To test the role of ATF4 in increasing mtDNA levels during infection, we generated ATF4 knockout (KO) ES-2 cells using CRISPR-Cas9 technology (Fig. 1G). The loss of ATF4 prevented the increase in mtDNA copy number during both *Toxoplasma* infection or tunicamycin treatment in wild-type (WT) cells (Fig. 1H-J; Fig. S5). Thus, ATF4 activation regulates mtDNA levels during infection and infection-independent stress.

## ATF4 increases mtDNA levels in a mitochondrial 1C metabolism-dependent manner

How does ATF4 regulate mtDNA during infection? Because infection induced the expression of *TFAM* and *TWNK,* we posited that ATF4 regulated mtDNA levels in a TFAM- and TWNK-dependent manner (Fig. S4). To address this possibility, we examined their expression in uninfected and *Toxoplasma-*infected WT and ATF4 KO cells. Contrary to our expectation, the loss of ATF4 did not block the increase in *TFAM* and *TWNK* transcripts during infection (Fig. S4). In fact, ATF4 KO cells had higher expression of *TWNK* relative to WT uninfected and *Toxoplasma-*infected cells (Fig. S4A). These results indicated that ATF4 increases mtDNA levels independently of TFAM and Twinkle.

**Fig. 2.**
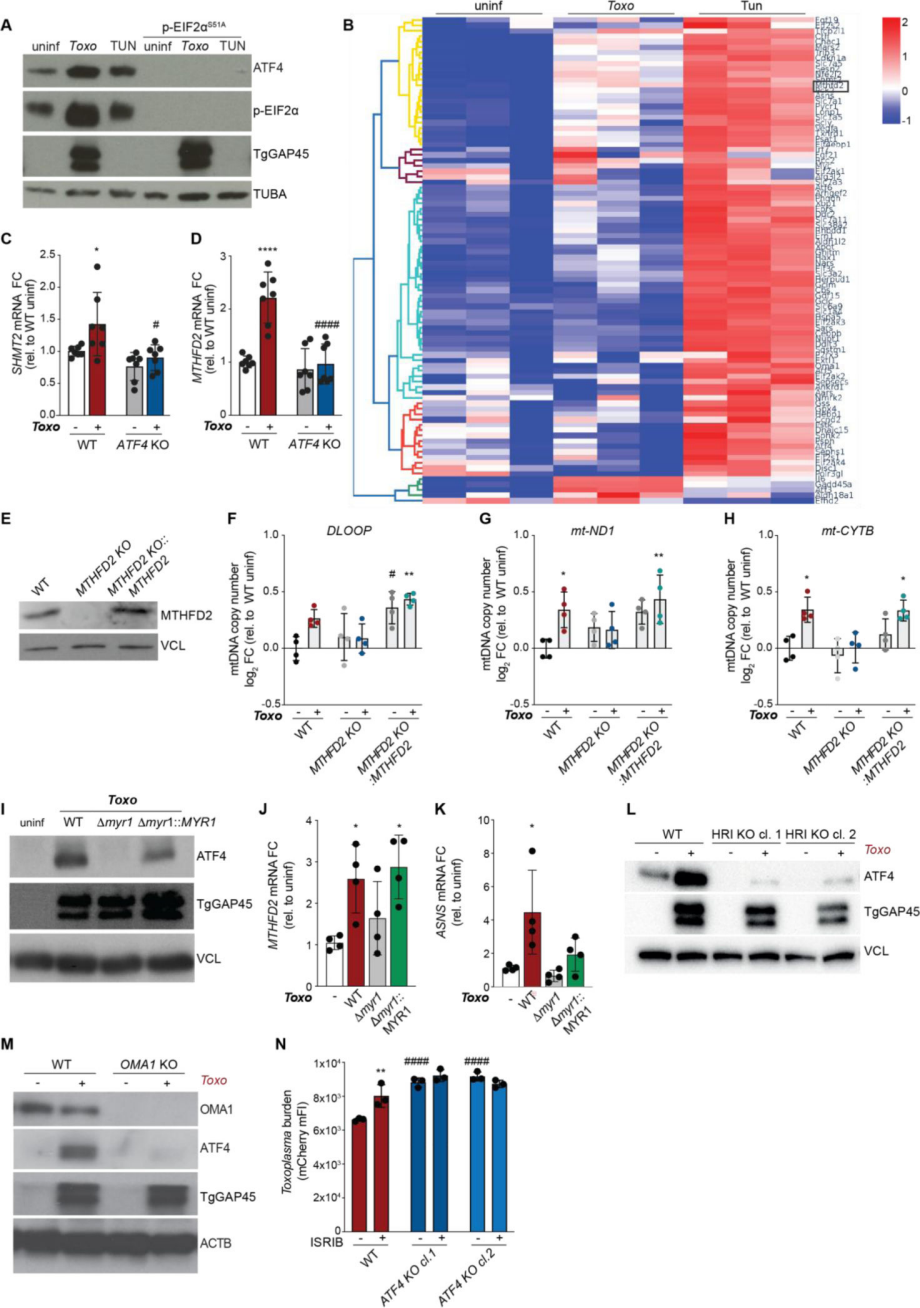
ATF4 requires mitochondrial one-carbon metabolism to increase mtDNA levels. (**A**) Immunoblot (IB) analyses of WT and eIF2α^S51A^ AN3–12 cells that were uninfected (uninf), infected with *Toxoplasma* (*Toxo*), or tunicamycin (TUN)-treated (3 μg/ml) for 24 hours (h): ATF4; phospho-EIF2α (Ser51), ~38 kDa; α-Tubulin (TUBA), ~55 kDa; and TgGAP45. (**B**) Heatmap of mRNA levels of *ATF4* target genes in uninf, *Toxo*, and TUN (3 μg/ml)-treated ES-2 cells; n=3 independent cultures. (**C** and **D**) WT and *ATF4* KO ES-2 cells were uninf or *Toxo* for 24 h and analyzed by qPCR for *SHMT2* and *MTHFD2*. Transcripts were normalized to *ACTB* and are relative to WT uninf. Data are mean ± SD of n=7 independent cultures. *p<0.05; ****p<0.0001 for uninf versus *Toxo* by two-way ANOVA analysis. (**E**) IB of ES-2 cells as indicated: MTHFD2, ~32 kDa and TUBA. (**F, G** and **H**) ES-2 cells as indicated were analyzed for mtDNA by qPCR for *DLOOP*, mt*-ND1* and mt*-CTYB* normalized to *RUNX2* levels and relative to WT uninfected. Data are mean ± SD of n=4 independent cultures. *p<0.05; **p<0.01 for uninf versus *Toxo*, and #p<0.05; ##p<0.01 for WT versus *MTHFD2* KO by two-way ANOVA analysis. (**I**) IB analysis of ES-2 cells as indicated at 24 hpi (MOI: 4 for *Toxo*): ATF4, VCL, and TgGAP45. (**J** and **K)** Cells treated as in (**I**) were analyzed by qPCR as in (**C** and **D**). Data are mean ± SD of n=4 independent cultures, *p<0.05; **p<0.01 by one-way ANOVA analysis. (**L**) IB analysis of WT and HRI KO ES-2 cells that were uninf or *Toxo* at 24 hpi; *Toxoplasma* GRA7 (TgGRA7), ~32 kDa, (**M**) IB analysis of uninf and *Toxo* WT and OMA1 KO mouse embryonic fibroblasts (MEFs) at 24 hpi: OMA1, ~35 kDa, ATF4, ACTB, and TgGAP45. (**N**) *Toxo* WT and *ATF4* KO ES-2 were treated ± ISRIB (200nM) and analyzed 24 hpi via flow cytometry for *Toxoplasma* burden (mCherry median FI). Data are mean ± SEM of n=3 biological experiments, **p< 0.01 for DMSO versus ISRIB, ####*p* < 0.0001 for WT versus ATF4 KO by two-way ANOVA analysis.

Because gene targets of ATF4 vary depending on its mechanism of activation, we next sought to define the host pathway upstream of ATF4 during infection (25). During the ISR, diverse cellular stresses are sensed by the so-called ISR kinases, which subsequently phosphorylate eIF2α at serine 51 (22). The consequent attenuation of translation leads to the translation of ISR effectors such as ATF4 (22). Alternatively, ATF4 can be activated by pro-growth signals that stimulate mTORC1, the anabolic regulator of the cell (26). To determine whether the ISR or mTORC1 mediated ATF4 activation during infection, we generated mouse embryonic stem cells cells (mESCs) deficient for eIF2α phosphorylation (eIF2α^S51A^). In WT mESCs, both *Toxoplasma* infection and tunicamycin treatment drove eIF2α phosphorylation, and ATF4 induction as in ES-2 cells (Fig. 2A, Fig. S7A). However, neither induced ATF4 in eIF2α^S51A^ mESCs or in ES-2 cells treated with ISRIB, a small molecule inhibitor of the ISR (Fig. 2A; Fig. S7A). Consistent with this result, rapamycin- or torin1-based inhibition of mTORC1, as assessed by phosphorylation of its target ribosomal protein S6 kinase 1 (S6K1), did not block ATF4 activation during *Toxoplasma* infection (Fig. S7B). Thus, infection activates ATF4 in an ISR-dependent manner.

To identify the ATF4 targets common to infection and ISR activation that mediated the increase in mtDNA levels, we compared the expression of a panel of ATF4 targets in uninfected cells, *Toxoplasma-*infected cells, and cells treated with tunicamycin (Table S2) (27). ATF4 targets induced by both *Toxoplasma* infection and tunicamycin treatment included the transcription factor *ATF3* and asparagine synthase (*ASNS*) (Fig. 2B). Of particular interest were *MTHFD2* and *SHMT2*, enzymes of the mitochondrial arm of the 1C metabolism processes that use folate as cofactor to activate and transfer 1C groups for the biosynthesis of nucleotide precursors (Fig. 2B) (7, 23, 27, 28). Furthermore, MTHFD2 has been linked to mtDNA deficiencies (8, 28). As expected, infection induced *MTHFD2* and *SHMT2* in an ATF4-dependent manner (Fig. 2C-D). To test the importance of mitochondrial 1C (mito-1C) metabolism in the changes in mtDNA during infection, we generated cells deficient for MTHFD2 (Fig. 2E). Infection did not increase mtDNA levels in MTHFD2 KO cells, unlike in WT cells (Fig. 2F-H). The overexpression of *MTHFD2* cDNA in MTHFD2 KO cells rescued the increase in mtDNA levels in infected cells, and even increased mtDNA levels in uninfected cells (Fig. 2F-H). Thus, mito-1C metabolism is required for ATF4 to increase mtDNA levels during infection.

## Host cells activate ATF4 in response to mitochondrial stress induced by parasite effectors

Because ATF4 regulated mtDNA dynamics in a mito-1C metabolism-dependent manner, we hypothesized that the activation of ATF4 comprised a defensive host response to *Toxoplasma.* However, the possibility remained that the ISR was activated by metabolic stress caused by the siphoning of nutrients by replicating parasites, as previously suggested (19). To distinguish between these possibilities, we first tested if the induction of ATF4 required parasite effector proteins that are secreted into the host cell. To do so, we examined ATF4 activation in cells infected with Δ*myr1* parasites that lack the translocon apparatus required for the secretion of a class of effector proteins, but are competent for replication and thus nutrient siphoning (29, 30). Host cells infected with Δ*myr1* parasites did not induce ATF4 nor its targets *MTHFD2* and *ASNS* (Fig. 2I-K; Fig. S6D). In line with this result, infection similarly induced ATF4 activation in cells deficient for the nutrient-sensing ISR kinase GCN2 and their WT counterparts (Fig. S8A-B). Thus, host cells activate ATF4 in response to a secreted parasite effector.

How are *Toxoplasma* effectors sensed by the ISR? Having excluded a role for GCN2, we next asked whether the ISR kinases (PKR)-like endoplasmic reticulum kinase (PERK), Protein kinase R (PKR), or eukaryotic translation initiation factor 2-alpha kinase 1 (HRI) mediated ISR activation during infection (22). Infection did not induce levels of BiP, a canonical marker of ER stress that is sensed by PERK, unlike the ER stressor tunicamycin (Fig. S7A). PKR is mainly activated by double-stranded RNA during viral infection (22). We thus focused on HRI, which relays mitochondrial stress to the ISR (31). To this end, we generated HRI KO cells, which, as expected, had impaired ATF4 induction following treatment with the ATP synthase inhibitor oligomycin (Fig. S8C). HRI was required for the induction of ATF4 during infection (Fig. 2L). To further understand how infection induces the ISR, we tested the role of OMA1, a stress-activated mitochondrial protease that mediates HRI activation through its cleavage of the inner mitochondrial membrane protein DELE1 (31). As with HRI KO cells, OMA1 KO cells were deficient for the activation of ATF4 during infection (Fig. 2M). Thus, HRI activates the ISR in response to parasite effector-induced mitochondrial stress.

Having established that host cells activate the ISR in response to effector-induced mitochondrial stress, we next asked whether the ISR benefited host cells or *Toxoplasma*. Indeed, both the pharmacological suppression of the ISR via ISRIB and the genetic ablation of ATF4 led to significant increases in parasite replication (Fig. 2N; Fig. S7A). Furthermore, the expression of wild-type ATF4, but not a DNA-binding ATF4 mutant, was sufficient to restrict *Toxoplasma* growth in ATF4 KO cells (Fig. S9). Thus, the activation of ATF4 in response to host detection of *Toxoplasma* effectors restricts parasite growth.

## ATF4 drives mitochondrial 1C-metabolism and limits parasite dTMP synthesis and proliferation

**Fig. 3.**
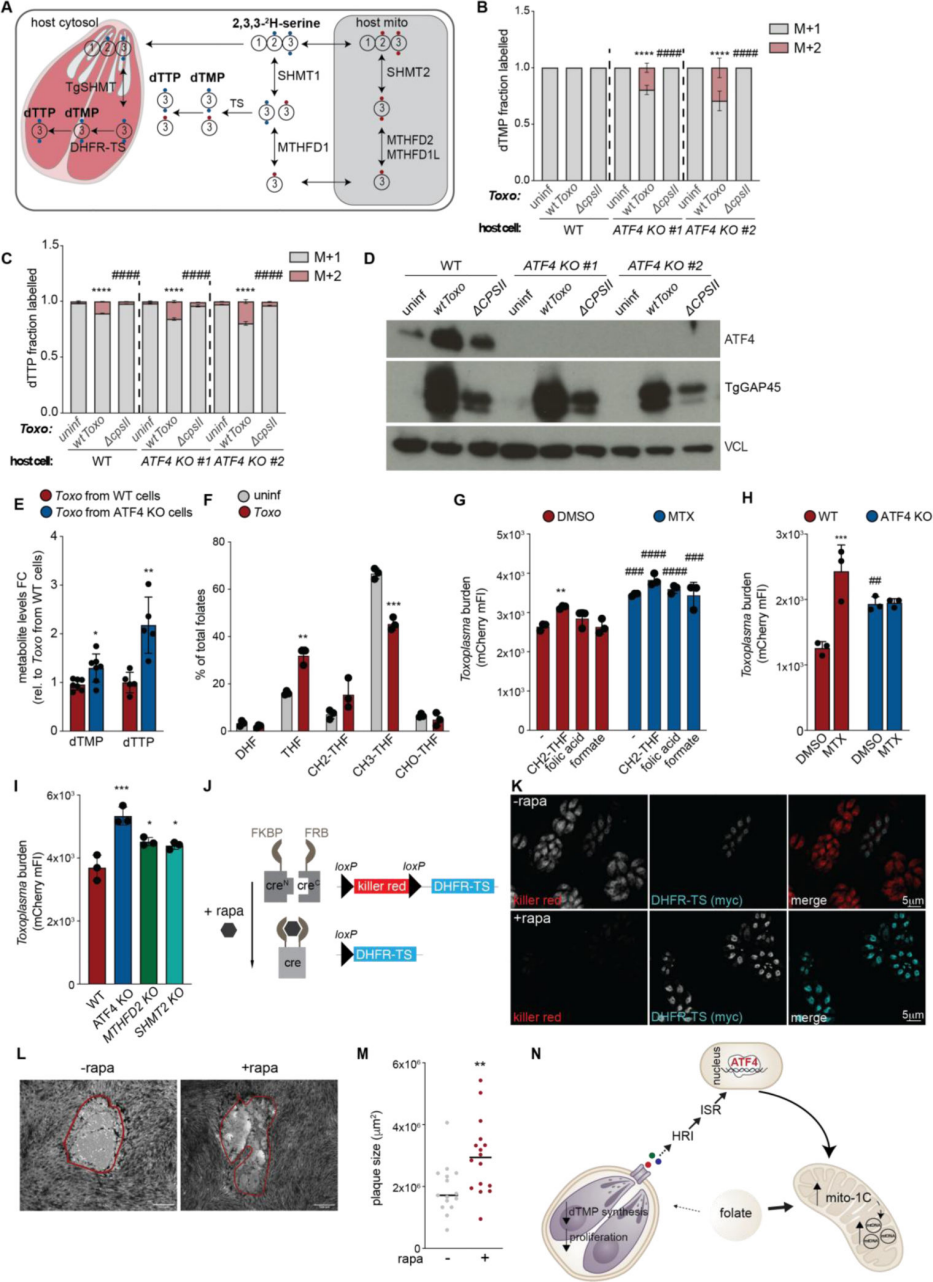
Mitochondrial 1C metabolism restricts parasite dTMP synthesis and replication. (**A**) M+2 ^2^H-labelleled dTMP from cyto-1C (2 blue dots), M+1 ^2^H-labelled dTMP from mito-1C (1 red dot). (**B** and **C**) Labeled dTMP and dTTP in WT and *ATF4 KO* ES-2 cells uninfected (uninf) or infected (inf) with WT *Toxo* and Δ*cpsII* parasites. Data are mean ± SD of n=3; ****p<0.0001 for uninf vs. inf ####p<0.0001 for WT vs. ATF4 KO clones by two-way ANOVA. (**D**) ATF4, VCL, and TgGAP45 in ES-2 cells as indicated. (**E**) dTMP levels in *Toxo* from WT and *ATF4* KO cells. Data are mean ± SD of n=7, dTTP not detected in two cultures; *p<0.05; **p<0.01; for WT vs. *ATF4* KO by unpaired t-test. (**F**) Total folate species (%) in uninf and inf ES-2 cells in minimal essential media, normalized to cell number. DHF: dihydrofolate; CH2-THF: 5,10-methylene-tetrahydrofolate; CH3-THF: 5-methyl-tetrahydrofolate; CHO-THF: 10-formyl-tetrahydrofolate. Data are mean ± SD of n=4; **p<0.01; ***p<0.001 for uninf vs. inf by multiple unpaired t-test. (**G**) Inf WT ES-2 cells treated ± MTX (200 nM) and either 68 μM folic acid; 2mM formate, or 25μM CH3-THF, and analyzed by flow cytometry for *Toxoplasma* mCherry median FI. Data are mean ± SEM of n=3; **p < 0.001 for untreated vs. folate(s), ###*p* < 0.001, ####p<0.0001 for DMSO vs. MTX by two-way ANOVA. (**H**) ES-2 cells treated ± MTX (200 nM) and analyzed as in (G). Data are mean ± SEM of n=3; ***p < 0.001 for DMSO vs. MTX; ##*p*< 0.01 for WT vs. ATF4 KO by two-way ANOVA analysis. (**I**) ES-2 cells as indicated analyzed as in (**G**). (**B-I**) Harvested at 24 hpi. Data are mean ± SEM of n=3; *p<0.05; ***p< 0.001 by one-way ANOVA. (**J)** Inducible DHFR-TS system. (**K**) IF images of HFFs inf with Di-Cre parasites expressing killer-red (-rapa) or DHFR-TS (+rapa). (**L-M**) Plaque assays and quantification of Di-Cre parasites ± rapa (50nM) in HFFs at 7 dpi, n=15; **p<0.01 by unpaired t-test. (**N**) Model of ATF4-drived folate-based immunity.

How does ATF4 affect mitochondrial metabolism and parasite growth? To address this question, we first assessed the contribution of mito-1C metabolism to dTMP and dTTP biosynthesis during *Toxoplasma* infection. Because serine is the predominant donor of carbons to 1C pathways in cultured cells, we cultured uninfected and *Toxoplasma-*infected WT cells with deuterium-labelled serine [2,3,3-^2^H-serine] and examined the resulting incorporation of ^2^H into dTMP or dTTP (Fig. 3A) (7, 32). The contribution of mito-1C-derived folates leads to dTMP and dTTP with 1 deuterium and a mass shift of 1Da (M+1); the contribution of cytosolic-1C (cyto-1C)-derived folates will instead lead to the incorporation of 2 deuteriums and results in M+2 dTMP or dTTP (Fig. 3A) (32). In uninfected WT cells, M+1 dTMP and M+1 dTTP were the dominant isotopologues at 100% and >98% of their respective isotope-labeled pools (Fig. 3B-C). This result is in line with previous observations that mito-1C predominately supplies 1C units in most cultured cell lines (32). However, *Toxoplasma* infection of WT cells led to a 10x-fold increase in M+2 dTTP levels. M+2 dTMP was not detected, likely due to the lower abundance of intracellular dTMP pools (Fig. 3B-C). We next assessed the effect of ATF4 ablation on mito-1C metabolism during infection. The loss of ATF4 led to a significant increase in the contribution of non-mito1C metabolism to dTMP and dTTP synthesis. M+2 dTMP, which was not detected in WT infected cells nor uninfected ATF4 KO cells, made up ~20% of labelled dTMP in infected ATF4 KO cells, while M+2 dTTP was ~2x-fold higher than infected WT cells (Fig. 3B-C). Thus, ATF4 maintains host mito-1C metabolism during infection.

We reasoned that the observed increase in M+2 dTMP and dTTP in infected WT and ATF4 KO cells could result from a shift to host reliance on host cyto-1C. Alternatively, because *Toxoplasma* expresses a single cytosolic SHMT and can synthesize pyrimidines de novo, parasite-derived dTMP and dTTP synthesis could also contribute to the increase in M+2 dTMP and dTTP isotopologues (Fig. 3A) (33, 34). To distinguish between these possibilities, we first assessed levels of pyrimidines in uninfected and *Toxoplasma-*infected cells treated with or without leflunomide, a small molecule inhibitor of host dihydroorotate dehydrogenase and thus pyrimidine synthesis (35). Infection led to ~10x-fold and ~2x-fold increases in total dTMP and dTTP, even in the presence of leflunomide, despite the expected accumulation of the pyrimidine precursors carbamoyl aspartic acid and dihydroorotic acid (Fig. S10A-B). Thus, the increases observed during infection were, in part, driven by *Toxoplasma*-derived dTMP and dTTP.

We next addressed whether the increase in M+2 dTMP and dTTP in ATF4 KO cells was due to their synthesis by host cells or *Toxoplasma*. To do so, we examined M+1 and M+2 isotopologues of dTMP and dTTP during infection with parasites that are deficient for their de novo synthesis due to the loss of carbamoyl phosphate synthetase II (CPSII), and thus only replicate if supplemented with exogenous uracil (33). Infection with *ΔcpsII* parasites led to ATF4 activation, but unlike in the case of WT parasites, failed to drive the increases in M+2 dTMP and M+2 dTTP observed in ATF4 KO cells (Fig. 3B-D). Thus, the M+2 dTMP and M+2 dTTP detected in infected ATF4 KO cells were parasite-derived, and ATF4 loss enhances parasite dTMP synthesis. Consistent with this, parasites isolated from ATF4 KO cells had higher total dTMP and dTTP levels compared WT cells, but similar levels of host-derived dTMP (Fig. 3E; Fig. S11). Moreover, blocking host 1C metabolism with the DHFR inhibitor methotrexate (MTX) led to the expected loss of host M+1 dTMP and dTTP synthesis, but drove ~2x-fold increases in parasite M+2 dTMP and dTTP levels (Fig. S12). Thus, ATF4-induced mito1C restricts parasite dTMP synthesis.

How does ATF4 activation regulate parasite dTMP levels? Unlike in mammals, in *Toxoplasma,* the synthesis of dTMP is catalyzed by the bifunctional enzyme dihydrofolate reductase thymidylate synthase (DHFR-TS) (Fig. S13A) (36). This means that parasites couple the generation of dTMP, the rate-limiting step in DNA synthesis, to the reduction of dihydrofolate (DHF) to tetrahydrofolate (THF). To synthesize dTMP, *Toxoplasma* requires 5, 10 methylene-THF (CH2-THF) and the ability to reduce DHF, a byproduct of this reaction, back to THF—the form capable of carrying 1C units and that is necessary for regenerating CH2-THF (Fig. S13A). Because ATF4 activation induced increased mito-1C cycle activity that uses 1C-carrying folates, we reasoned that ATF4 restricted *Toxoplasma* access to the folates required for dTMP synthesis and parasite growth. We thus assessed levels of folates in uninfected and infected cells using mass spectrometry. Infection caused a marked reduction in CH3-THF—the main circulating form of folate—and total folate levels, indicating that 1C cycle activity is disrupted during infection and may restrict the availability of key folates (Fig. 3F; Fig. S13B-C). Indeed, supplementation with CH2-THF, which is required for *Toxoplasma* dTMP synthesis but not its precursor folate nor derivative formate, promoted parasite growth regardless of host 1C activity (Fig. 3G; Fig. S13A). Thus, ATF4 restricts *Toxoplasma* access to folates required for dTMP synthesis.

To test whether ATF4 restricted parasite growth in a folate-dependent manner, we treated WT and ATF4 KO cells with MTX. MTX is not taken up by *Toxoplasma,* and thus does not inhibit *Toxoplasma* DHFR-TS (37). MTX treatment increased parasite proliferation in WT cells, but not in ATF4 KO cells (Fig. 3H). Furthermore, impairing mito-1C metabolism through ablation of the ATF4 target MTHFD2, SHMT2, or knockdown of the reduced folate carrier SLC19A1 rendered cells permissive to *Toxoplasma* replication and increased dTMP levels (Fig. 3I, Fig. S14) (38). Thus, ATF4 restricts parasite growth in a mito-1C-dependent manner.

If dTMP is rate-limiting for *Toxoplasma* growth, we expected that increasing its synthesis should promote *Toxoplasma* growth. To test this possibility, we turned to *Toxoplasma* DiCre (dimerizable Cre recombinase) parasites for the inducible overexpression of DHFR-TS (39). In these parasites, Cre recombinase is expressed as two inactive fragments that are fused to rapamycin binding proteins FRB and FKBP (Fig. 3J) (39). DiCre parasites were engineered to constitutively express killer red unless cre-mediated recombination occurs, in which case myc-tagged DHFR-TS is expressed (Fig. 3J-K). In monolayers of human fibroblasts, rapamycin-induced DHFR-TS-expressing parasites formed significantly larger plaques than killer red-expressing parasites; of note, rapamycin did not affect the growth of WT parasites (Fig. 3K-M). Thus, dTMP synthesis is rate-limiting for *Toxoplasma* growth. Furthermore, ATF4 rewires mitochondrial metabolism to sequester 1C-carrying folates from *Toxoplasma* (Fig. 3N).

## ATF4 activates a host-protective response in vivo

**Fig. 4.**
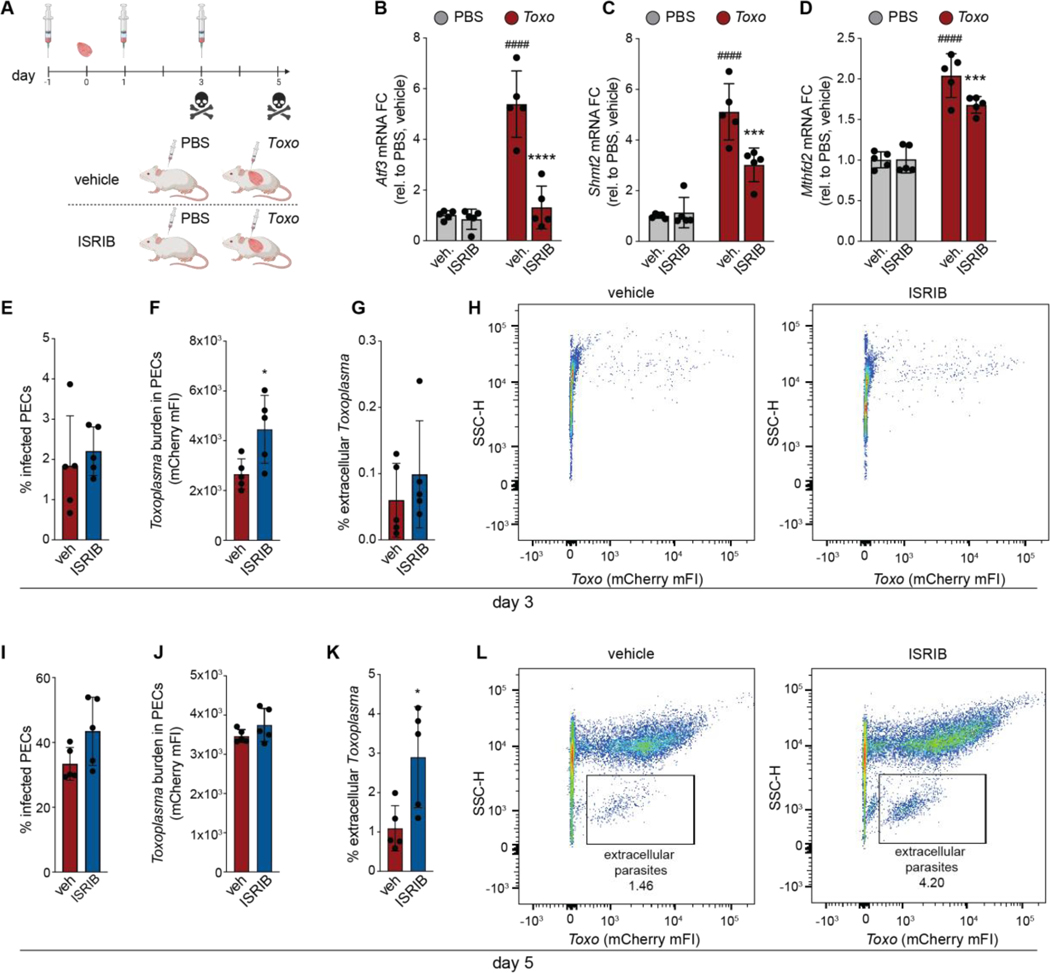
ATF4 protects against *Toxoplasma* infection in vivo. (**A**) Schematic of infection and ISRIB administration. Mice (n=5) where injected with ISRIB one day before infection with 50 tachyzoites or 1x PBS injection and every other day after (**B-D**) Peritoneal exudate cells (PECs) were isolated from mice treated as in (**A**) at 5 days post injection (dpi) and analyzed for the indicated ATF4 target genes by qPCR. Transcripts were normalized to *Hprt* and relative to uninfected PECs mice (n=5). ***p<0.001; ****p<0.0001 for vehicle versus ISRIB and #p < 0.05; ###p < 0.001; and ###p < 0.0001 for PBS versus *Toxoplasma* by means of two-way ANOVA analysis. (**E**) Peritoneal exudate was isolated 3 dpi from mice treated as in (**A**) and analyzed by means of flow cytometry for (**E**) % infected PECS; (**F)** *Toxoplasma* parasite burden in infected PECs (mCherry median FI) and (**G**) % of extracellular parasites. Data are mean ± SD of n=5 mice, *p<0.05 by unpaired t-test analysis (vehicle vs. ISRIB). (**H**) Representative scatter plots of peritoneal exudate isolated from mice treated as in (**A**). (**I**-**L**) Same as E-H except at 5 dpi.

We next asked whether ATF4 activated a host-protective response in vivo. To address this question, we first asked whether ATF4 was activated in a mouse model of *Toxoplasma* infection. We injected mice intraperitoneally with vehicle, tunicamycin, or mCherry-expressing *Toxoplasma* and measured the levels of ATF4 target transcripts in peritoneal exudate cells (PECs) isolated at 5 days post infection (dpi), near the peak of acute infection (40). Infection drove increases in the levels of ATF4 targets *Atf3, Shmt2*, and *Mthfd2*, similar to our results in cultured cells (Fig. 2B; Fig. S15A-D). To determine whether the observed increases during infection were due to the ISR, mice were injected with vehicle or the ISR inhibitor ISRIB prior to infection and after every other day (Fig. 4A). ISRIB treatment blunted the induction of ATF4 targets *Atf3, Mthfd2* and *Shmt2* levels observed in PECs isolated from infected mice (Fig. 4B-D). We next tested whether blocking the ISR affected *Toxoplasma* proliferation in vivo. To this end, we examined the progression of *Toxoplasma* infection by examining peritoneal exudate of ISRIB- versus vehicle-treated mice at 3 dpi and 5 dpi. ISRIB treatment led to higher intracellular *Toxoplasma* levels as assessed at 3 dpi, which corresponded with an ~3-fold increase in extracellular parasites at 5 dpi (Fig. 4E-L). Similar results were obtained at 5 dpi and 7 dpi with Type II parasites (Fig. S16). Thus, activation of the ISR restricts parasite proliferation in vivo.

## Conclusions

Here we have shown that ATF4 effects a folate-based metabolic defense against *Toxoplasma* that involves increased activity of mitochondrial-1C metabolism. This raises several questions, beginning with how does HRI sense *Toxoplasma* effectors to activate the ISR? Both OMA1 and HRI were required for the secretion of effector proteins to trigger ATF4 induction. A *Toxoplasma* effector(s) could thus directly activate OMA1 or induce a mitochondrial stress that drives OMA1 cleavage of DELE1 and its subsequent activation of HRI in the cytosol.

How is mito-1C metabolism host-protective? Although *Toxoplasma* is capable of folate synthesis, it also scavenges exogenous folate, suggesting this metabolite is required in abundance for parasite proliferation (41). Unlike in mammals, in *Toxoplasma* folate availability is tied to the production of dTTP, the rate-limiting step in DNA synthesis. Because *Toxoplasma* is deficient for thymidine salvage, it likely that the parasite depends on DHFR-TS for dTTP synthesis and thus DNA replication *(12)*. Compartmentalization of 1C metabolism processes within the mitochondria might block parasite access to the 1C-carrying folates required for dTMP synthesis. Indeed, supplementation with CH2-THF increased parasite growth while the loss of ATF4 led to an increase in parasite dTMP synthesis and proliferation. Furthermore, impairing host use of folate for 1C-metabolism processes via MTX, a drug that is widely used in anti-cancer therapies, led to increased growth in WT cells but not ATF4 KO cells. Although these results support a role for mito-1C metabolism in limiting parasite access to folate, we cannot exclude the possibility that ATF4 activates other 1C-effectors that contribute to *Toxoplasma* restriction.

Can enhancing host mito-1C suppress the growth of *Toxoplasma* in vivo, or other human pathogens that require folate for dTMP synthesis such as *Plasmodium falciparum,* the causative agent of malaria? Conversely, does increasing folate availability through dietary supplementation without a parallel modulation of mito-1C metabolism enhance replication of these pathogens? High dietary folate increased parasite replication in a murine malaria model, and increased folate concentrations were associated with an increase in malaria infection in humans (42, 43). These observations hint at a key role for folate competition in certain host-pathogen interactions.

Finally, do mitochondria more broadly compete with intracellular pathogens for metabolites? Emerging evidence shows that the microbiota protect against gut infections by consuming nutrients also used by pathogens (44). Analogously, mitochondria may contribute to cellular resistance to infection by competing with intracellular pathogens for shared cytosolic resources beyond 1C-carrying folates, such as nucleotides. Although the demand to replicate a single 16 kB mitochondrial genome may seem minimal, each of the estimated ~880 mitochondria per cell contains on average 3 genomes—adding up to ~42 MB of total mtDNA (1). Indeed, during chronic mito-1C activation due to mtDNA deficiency, mitochondria even limited the availability of nucleotides for nuclear DNA replication (9, 10).

Our discovery of ATF4 as an effector of a folate-driven metabolic defense response reveals a role for mitochondrial metabolism in defense against the human pathogen *Toxoplasma*. These findings establish a framework for exploring the role of folate metabolism during infection, and noncanonical mitochondrial defenses against intracellular pathogens.

## Materials and Methods

### Cell culture and cell lines

HeLa adenocarcinoma cells, ES-2 ovary clear cell carcinoma, Human foreskin fibroblasts (HFF), were obtained from ATCC (CCL-2, CRL-1978, and SCRC-1041, respectively); ES-2 cells stably expressing pMXs-eGFP-OMP25 (referred to as OMM-GFP; Addgene #83356) were generated through lentiviral transduction. Unless stated otherwise cell lines were cultured in Dulbecco’s Modified Eagle’s Medium (DMEM) containing 10% heat-inactivated fetal bovine serum (FBS) and maintained at 37°C and 5% CO_2_. AN3–12 mouse embryonic stem cells were obtained from Dr. Josef Penninger (45) and were cultured in DMEM high glucose supplemented with glutamine, 15% fetal bovine serum, penicillin/streptomycin, nonessential amino acids, sodium pyruvate, β-mercaptoethanol, and LIF as previously described (46). *ATF4* KO ES-2s were cultured in DMEM high glucose, 10% fetal bovine serum, β-mercaptoethanol and nonessential amino acids. Cells were tested every 2 weeks for *Mycoplasma* infection by means of polymerase chain reaction (PCR). Immortalized WT and *Oma1* KO murine embryonic fibroblasts (MEFs) were obtained from Thomas Langer lab and cultured in Dulbecco’s Modified Eagle’s Medium (DMEM) containing 10% heat-inactivated fetal bovine serum (FBS) (47).

### Parasite culture and strains

*Toxoplasma gondii* parasites of the Type I RHΔ*ku80:mCherry+* (previously described in (48)); Type I RHΔ*myr1* parasites and RHΔ*myr1::Myr1* were provided by Dr. Moritz Treeck, (Gulbenkian Institute) (49); Type I RHΔcpsII:mCherry parasites were provided by Dr. David Bzik (Dartmouth Geisel School of Medicine) (33). Type II Me49Δmyr1 *Toxoplasma gondii* parasites were provided by Dr. John Boothroyd (Stanford School of Medicine). Parasites were maintained by serial passage in human foreskin fibroblast (HFF) monolayers in cDMEM. RHΔ*cpsII*: mCherry were maintained by serial passage in human foreskin fibroblast (HFF) monolayers in cDMEM supplemented with 0.2 mM uracil. DiCre parasites were provided by Dr. Marcus Meissner (39) Generation of DiCre Type I parasites inducibly expressing DHFR-TS: To remove/insert needed restriction sites, KillerRed was amplified from the previously generated loxP-KillerRed-loxP-ATrX2 and re-inserted between EcoRI and NsiI to remove the AvrII site and add an additional MfeI site (50). TGGT1_249180 was amplified from *Toxoplasma* cDNA and was cloned in between MfeI and AvrII sites replacing ATrX2. TUB8-loxP-KillerRed-loxP was amplified from the original loxP-KillerRed-loxP-ATrX2 vector and re-inserted between ApaI and MfeI sites. RH DiCre parasites were transfected with 50μg of the resulting plasmid. Positive transfectants were selected with mycophenolic acid (25 mg/mL) and xanthine (50 mg/mL) and cloned out. Clonal line was verified with IFA.

### Plaque assay

100 of either Type I RHΔ*ku80:mCherry+* or RH DiCre: loxP-KillerRed-loxP-DHFR-TS were added to monolayers of confluent foreskin fibroblasts (HFFs) ± rapa (50nM). At 7 dpi, monolayers were incubated with 100% MeOH for 1 min, followed by 20 minutes with crystal violet.

### CRISPR/Cas9-mediated gene editing

Guide sequences used can be found in Table S3. ATF4, MTHFD2, SHMT2 knockout (KO) cells and SLC19A1 knockdown (KD) cells were generated using CRISPR‐Cas9 mediated gene editing via lentiviral transductions into the pLenti CRISPRv2 (Addgene #5296). HRI and GCN2 KO were generated using CRISPR‐Cas9 mediated gene editing with the pLenti CRISPRv2 EIF2AK1/HRI (Addgene #218529) and the pLenti CRISPRv2 EIF2AK1/HRI (Addgene #218528), respectively. For production of lentiviral particles, 293T human embryonic kidney (HEK) cells were transduced using the X-tremeGENE 9 DNA Transfection Reagent (Roche) with 1 μg psPAX2 packaging vector (Addgene #12260), 0.3 μg pCMV-VSVG envelope vector (Addgene #8454) and 1 μg of the relevant plasmid of interest. The next day, the medium was exchanged, and two days post transfection, the virus-containing supernatant was filtered with a 0.45 μM filter and supplemented with polybrene to a final concentration of 5 μg/ml. Virus‐containing medium was added to the indicated cell lines for 24h and puromycin selection (3 μg/ml) was started after an additional 24–48 h. Polyclonal cultures and individual clones were validated by immunoblotting or qPCR.

The eIF2α S51A substitution was engineered in AN3–12 cells using CRISPR/Cas9 technology as described previously (51). DNA template sequences for small guide RNAs were designed online (http://crispor.org), and cloned into the Cas9-mCherry expressing plasmid (Addgene #21852). Corresponding guide and Cas9 expressing plasmids were co-transfected with a single-stranded DNA repair template (Integrated DNA Technologies), using Lipofectamine 2000 (Thermo Fisher Scientific) according to the manufacturer’s instructions. mCherry-positive cells were singled in 96-well plates using FACSAria Fusion sorter and subjected to genotyping. DNA was extracted (DNA extraction solution, Epicentre Biotechnologies) and edited regions were specifically amplified by PCR. The PCR product was subjected to a restriction digest to identify positive clones. These clones have a gain of BglII restriction site due to a mutation in the repair template. Sanger sequencing was performed at Eurofins Genomics GmbH (Ebersberg, Germany). Positive clones were sorted prior to further experiments.

### *MTHFD2* and *ATF4 overexpression*

Human *MTHFD2* cDNA amplified from ES-2 cells was cloned into pMSCV PIG (puro IRES GFP; Addgene #21654) at the XhoI and EcoRI sites. Following production of viral particles and transduction, puromycin selected ES-2 cells were sorted for GFP expression using flow cytometry using BD FACSDiva software. The pTRIPZ-EGFP, pTRIPZ-ATF4, pTRIPZ-ATF4DBD plasmids were a gift from Dr. Manning (Harvard T.H. Chan Public School of Health) (25). HEK-293T cells were co-transfected with the given pTRIPZ plasmid, the VSV-G envelope plasmid and the ΔVPR lentiviral packaging plasmid using XTremeGene 9 Transfection Reagent (Roche). Following viral production and trasduction, cells were selected for puromycin. cDNA expression was induced with 1 μg/mL of doxycycline 24 hr before assays were conducted.

### Flow cytometry analysis

To assess mitochondrial mass, monolayers of ES-2 cells expressing OMM-GFP were rinsed with PBS, trypsinized and fixed in 2% paraformaldehyde in FACS buffer (3% FBS in 1XPBS) for 10 min. After a brief spin, cells were resuspended in FACS buffer and 10,000 events were analyzed on a BD LSRFortessa for GFP median fluorescence intensity (mFI) using BD FACSDiva software.

To assess parasite proliferation, monolayers of ES-2 cells infected with RFP expression parasites (RHΔku80:mCherry+) were rinsed with PBS, trypsinized and fixed in 2% paraformaldehyde in FACS buffer (3% FBS in PBS) for 10 min. After a brief spin, cells were resuspended in FACS buffer and 10.000 events analyzed on a LSRFortessa and the RFP mean fluorescence intensity (mFI) using BD FACSDiva software.

### Quantitative PCR for mtDNA analysis

For mtDNA measurements, genomic DNA was isolated from cell pellets that were washed once with 1x PBS using the Blood and Tissue DNA extraction kit (Qiagen #69504). DNA was quantified by nanodrop. 50 ng of genomic DNA were amplified using PowerSYBR Green PCR Master Mix (Thermo Fisher Scientific #A25742). Reactions were performed in 384 Hard-Shell microplates (BioRAD #HSP3801) sealed with adhesive qPCR seal (Roche) in a and Bio-Rad Real-Time PCR system with the following cycler program: 95°C for 7 min followed by 35 cycles of 95°C for 10 s, 60°C for 30 s. Dissociation curves confirmed single PCR products, and signals were analyzed within the linear amplification range. Each sample was run in triplicate. Relative mtDNA copy number changes were calculated using the comparative ΔΔCt method (52) by determining Ct threshold values and using equations ΔΔCt = (Ct_mtDNA − Ct_control) at *t*(*n*) − (Ct_mtDNA − Ct_control) at *t*(0) and 2^−ΔΔCt^. The primers *DLOOP, mt-ND1, mt-CYTB, mt-ND6 and mt-COX1* as mitochondrial genes and *RUNX2* and *ACTB* as nuclear DNA controls can be found in Table S3.

### Quantitative PCR for mRNA analysis

For analysis of mRNA expression, RNA purification was performed using TriZol (Thermo Fischer Scientific; #15596018) according to the manufacturer’s instructions. The Reverse Transcriptase reaction (RT) was done using 1 μg of RNA for each sample into cDNA using SuperScript™ VILO™ Master Mix (Thermo Fischer Scientific; # 11755050). For each reaction, 2.5 ng of cDNA were amplified using PowerSYBR Green PCR Master Mix and Bio-Rad Real-Time PCR system. For each independent sample, RT–qPCR was performed in technical triplicates. The primer sequences used in this study can be found in Table S3. Expression levels were calculated by the delta delta ct methods as described above, for which *ACTB* and *HPRT* was used as a control. Relative fold changes were calculated using the comparative ΔΔCt method (52) by determining Ct threshold values and using equations ΔΔCt = (Ct_targetgene − Ct_control) at *t*(*n*) − (Ct_targetgene − Ct_control) at *t*(0) and 2^−ΔΔCt^.

### Immunoblotting and antibodies

Whole cells were harvested in chilled lysis buffer (50mM Hepes-KOH pH 7.4, 40mM NaCl, 2mM EDTA, 1.5mM NaVO4, 50mM NaF, 10mM NaPyrophosphate, 10mM, NaBetaGlycerophosphate (disodium salt pentahydrate), 1% Triton X-100) and lysed for 30 min on ice. Lysates were subsequently centrifuged at 10 min at 14,000 x g at 4°C and the clarified supernatant was transferred into a fresh tube with 5X SDS for a final volume of 1X SDS. Following SDS-PAGE and gel transfer, membranes were blocked with TBS-0.05% Tween 20 (TBS-T) and 5% milk and the primary antibodies were incubated overnight. Following incubation, blots were washed three times in TBS-T and then incubated with horseradish peroxidase (HRP)-conjugated anti-mouse IgG (CST #7076) or anti-rabbit IgG (CST #7074) at a 1:4000 dilution for 45 minutes and developed using a chemiluminescence system (Pierce™ ECL Western Blotting Substrate or Pierce SuperSignal™ West Atto Ultimate Sensitivity Substrate; ThermoFisher Scientific). The following antibodies were used: ATF4 (CST #11815), VCL (CST #4650), pS6K (CST #97596), TUBA (Proteintech 66031–1-Ig), MTHFD2 (CST #41377), SHMT2 (CST #33443), MTHFD1L (Invitrogen #PA5–100158), MTHFD1 (Sigma #HPA015006), phospho-eIF2a (Ser51) (CST #9721), ACTB (CST #4970), OMA1 (Santa Cruz #515788), BIP (CST #3183), ATF6 (CST #65880), c-Myc ( CST #18583), anti-DNA (EMD Millipore #CBL186), GCN2 (CST#3302); GCN2 (abcam ab134053), TgGra45 (Dr. D Soldati; U. of Geneva), TgMAF1 and TgGRA7 (Dr. J Boothroyd; Stanford University) antisera.

### Live-cell imaging

Cells were plated on 6-well CELLview glass bottom cell culture dishes (Greiner Bio-One). The following morning, cells were infected with RH mcherry expressing *Toxoplasma*. After 24 hours, cells were incubated with MitoTracker Deep Red (Thermo Fisher Scientific, 50 nM) and Picogreen 1ug/ml for 30 min and imaged using an Olympus IXplore SpinSR 50 mm spinning disk confocal microscope. Live cell imaging was performed in cDMEM with incubation at 37°C and 5% CO_2_. All images were taken with a 100X/1.35 silicon oil objective and excitation with either 488, 561, or 640 laser lines, using ORCA-Flash4.0 cameras (Hamatsu), and cellSens Software.

### Edu replication assay

EdU incorporation was detected via Click-iT EdU AlexaFluor 488 labeling kit according to the manufacturer’s instructions (C10637, ThermoFisher) with minor deviations. For the EdU-labeling experiments, cells were processed for immunofluorescence analysis and stained with anti-DNA antibody.

### Immunofluorescence Assay

Cells were fixed using growth medium containing 3.7% PFA for 15 min at 37°C and permeabilized using 0.1% Triton X-100 in PBS for 20 min. Following incubation with 3% BSA for 1 hour, cells were incubated with primary antibodies (ATF4, 1:250) overnight. The next day, cells were washed 3X with 1X PBS (5 minutes each) before incubation with secondary antibodies for 1h in the dark at room temperature. Following secondary incubation, cells were cells were washed 3X with 1X PBS. All images were taken with a 100X/1.35 silicon oil objective and excitation with either 405, 488, or 561 laser lines, using ORCA-Flash4.0 cameras (Hamatsu), and cellSens Software.

### Proteomics sample preparation

As previously described in (53), for preparing samples from immunoprecipitation, on-beads digestion was performed to elute the proteins off the beads. Before adding the elution buffer, the beads were washed with detergent-free buffer (50mM Tris-HCl pH7.5) four times to remove any detergents used previously. Then 100 μl of the elution buffer (5ng/μl trypsin, 50mM Tris-HCl pH7.5, 1mM Tris(2-carboxyethyl)phosphine), 5mM chloroacetamide) was added to the beads and incubated at room temperature by vortexing from time to time, or rotating on a rotator. After 30 min, the supernatant was transferred to a 0.5 ml tube and incubated at 37°C overnight to ensure a complete tryptic digest. The digestion was stopped in the next morning by adding formic acid to the final concentration of 1%. The resulted peptides were cleaned with home-made StageTips. Alternatively, four micrograms of the eluted peptides were dried out and reconstituted in 9 μL of 0.1M TEAB and labeled with tandem mass tags (TMT10plex, Thermo Fisher Scientific cat. No 90110). Labeling was carried out according to manufacturer’s instruction with the following changes: 0.5 mg of TMT10plex reagent was re-suspended with 33 μL of anhydrous ACN. Seven microliters of TMT10plex reagent in ACN was added to 9 μL of clean peptide in 0.1M TEAB. The final ACN concentration was 43.75% and the ratio of peptides to TMT10plex reagent was 1:20. After 60 min of incubation the reaction was quenched with 2 μL of 5% hydroxylamine. Labelled peptides were pooled, dried, re-suspended in 200 μL of 0.1% formic acid (FA), split into two equal parts, and desalted using home-made STAGE tips. One of the two parts was fractionated on a 1 mm x 150 mm ACQUITY column, packed with 130 Å, 1.7 μm C18 particles (Waters cat. no SKU: 186006935), using an Ultimate 3000 UHPLC (Thermo Fisher Scientific). Peptides were separated at a flow of 30 μL/min with a 88 min segmented gradient from 1% to 50% buffer B for 85 min and from 50% to 95% buffer B for 3 min; buffer A was 5% ACN, 10mM ammonium bicarbonate (ABC), buffer B was 80% ACN, 10mM ABC. Fractions were collected every three minutes, pooled in two passes (1 + 17, 2 + 18 … etc.), and dried in a vacuum centrifuge (Eppendorf).

### LC-MS/MS analysis

For label-free quantification, peptides were separated on a 75 cm, 75 μm internal diameter Acclaim™ PepMap™ analytical column (Thermo Fisher Scientific, catalogue number 164939) using an EASY-nLC 1200 (Thermo Fisher Scientific). The column was maintained at 50°C. Buffer A and B were 0.1% formic acid in water and 0.1% formic acid in 80% acetonitrile. Peptides derived from whole cells were separated on a segmented gradient from 6% to 31% buffer B for 230 min and from 31% to 50% buffer B for 10 min at 250 nl / min. Peptides derived from enriched mitochondria were separated on a segmented gradient from 6% to 31% buffer B for 110 min and from 31% to 50% buffer B for 10 min at 250 nl / min. Eluting peptides were analyzed on a QExactive HF mass spectrometer (Thermo Fisher Scientific). Peptide precursor m/z measurements were carried out at 60000 resolution in the 300 to 1800 m/z range. The top ten most intense precursors with charge state from 2 to 7 only were selected for HCD fragmentation using 25% normalized collision energy. The m/z values of the peptide fragments were measured at 30000 resolution using a minimum AGC target of 1e4 and 55 ms maximum injection time for the whole cell samples or 15000 resolution, minimum AGS target of 1e4, and 120 ms maximum injection time for the enriched mitochondria samples. Upon fragmentation, precursors were put on a dynamic exclusion list for 45 sec. TMT labeled peptides were and separated on a 50 cm, 75 μm Acclaim PepMap column (Thermo Fisher Scientific, Product No. 164942) and analysed on a Orbitrap Lumos Tribrid mass spectrometer (Thermo Fisher Scientific) equipped with a FAIMS device (Thermo Fisher Scientific). The FAIMS device was operated in two compensation voltages, −50 V and −70 V. Synchronous precursor selection based MS3 was used for the acquisition of the TMT10plex reporter ion signals. Peptide separations were performed on an EASY-nLC1200 using a 90 min linear gradient from 6% to 31% buffer; buffer A was 0.1% FA, buffer B was 0.1% FA, 80% ACN. The analytical column was operated at 50°C. Raw files were split based on the FAIMS compensation voltage using FreeStyle (Thermo Fisher Scientific).

### Protein identification and quantification

Label-free quantification raw data were analyzed with MaxQuant version 1.6.0.13 (54) using the integrated Andromeda search engine (55). Peptide fragmentation spectra were searched against the canonical sequences of the human reference proteome, proteome ID UP000000589, and toxoplasma reference proteome, proteome ID UP000005641, downloaded from UniProt. Methionine oxidation and protein N-terminal acetylation were set as variable modifications; cysteine carbamidomethylation was set as fixed modification. The digestion parameters were set to “specific” and “Trypsin/P,” The minimum number of peptides and razor peptides for protein identification was 1; the minimum number of unique peptides was 0. Protein identification was performed at a peptide spectrum matches and protein false discovery rate of 0.01. The “second peptide” option was on. Successful identifications were transferred between the different raw files using the “Match between runs” option. Exploratory data analysis and visualization was done using tidyverse in R (56, 57). TMT data was analyzed using MaxQuant, version 1.6.17.0. The isotope purity correction factors, provided by the manufacturer, were included in the analysis. Differential expression analysis was performed using limma, in R (58). Volcano plots of WC and mitoIP proteins generated using Flaski (59). The mass spectrometry proteomics data have been deposited to the ProteomeXchange Consortium via the PRIDE (60) partner repository with the dataset identifier PXD064338; *token:Usy5DtrhLbiN*.

### Stable Isotope Labelling with L-glutamine-^15^N_2_

2 million ES-2 cells were seeded in a 10cm dishes in DMEM containing 10% FBS and cultured for 16 h. Next day cells were washed once with 1x PBS, and the medium was replaced with glutamine-free DMEM containing 10% dFBS and 2 mM *L-glutamine-*^*15*^*N*_*2*_ (Cambridge isotope laboratories #NLM-1328–0.25) for 24h. The following day, cells were either mock-infected or infected with *Toxoplasma (*RH*Δku80:mCherry+*) at a multiplicity of infection (MOI) of 4 in cDMEM media in the absence of labelled *L-glutamine-*^*15*^*N*_*2*_ for 24 hpi. Parasites were isolated from infected monolayers and processed for metabolomics analysis.

### Stable Isotope Labelling with L-serine-^13^C_3_

500.000 cells were seeded in a 6 well plate in DMEM containing 10% FBS. The following day, cells were either mock-infected or infected with *Toxoplasma (*RH*Δku80:mCherry+*) at a multiplicity of infection (MOI) of 4. After 12 hpi the medium was replaced with serine-free DMEM containing 10% dFBS and 0.2 mM L-serine-^13^C_3_ (Cambridge isotope laboratories #NLM-201595–68-8) for 10h. Mock infected and infected cells were then processed for metabolomics analysis.

### Stable Isotope Labelling with 2,3,3-^2^H-serine

WT and ATF4 KO ES-2 cells (250,000) were seeded in DMEM containing 10% FBS and cultured for 16 h. The following day cells were washed once with PBS, and either mock-infected or infected with *Toxoplasma* strains RH*Δku80:mCherry* and RH*ΔcpsII:mCherry* at a multiplicity of infection (MOI) of 4 and 6, respectively due to the lack of proliferation of RH*ΔcpsII:mCherry* parasites in minimal essential media (MEM) containing 10% dialyzed FBS, 2 mM L-glutamine and 0.2 mM [2,3,3-^2^H] serine isotopologue (Cambridge Isotope Laboratories #DLM-1073–1). At 24 hpi, cells were harvested for metabolomics analysis.

### Toxoplasma isolation for metabolite extraction

4 million cells ES-2 cells were seeded in 10 cm dishes overnight and infected with *Toxoplasma* at an MOI of 4 the subsequent morning. At 24 hpi, infected cells were collected by scraping and washed with ice-cold DPBS twice. All subsequent steps were performed at 4°C. Infected cells were resuspended in DPBS with phosphatase and proteases inhibitors and homogenized by passing 10 times through a 271/2 G needle. The homogenates were spun down at 1500 *rpm* for 5 min to remove cell debris and filtered with a 5 μM filter. The final pellets containing parasite-enriched fractions were resuspended in cold metabolite extraction buffer. The purity of the *Toxoplasma*‐enriched fractions was assessed by SDS‐PAGE and immunoblot.

### Extraction of polar metabolites

Cells were washed twice with 75 mM of ammonium carbonate pH = 7.4 and the plates were frozen in liquid nitrogen and stored at −80°C until metabolite extraction. For metabolite extraction, 1ml of −20°C cold extraction buffer (HPLC‐grade ultrapure 40% MeOH, 40% acetonitrile, 20% water, containing 250 nM ^13^C^15^N Amino acids (Cambridge isotopes MSK_A2–1.2), 100 ng/mL of ^13^C_10_ ATP (Sigma 710695), 100 ng/mL ^15^N_5_ ADP (Sigma 741167), 100 ng/mL ^13^C_10_^15^N_5_ AMP (Sigma 650676) and 20 ng/mL citric acid ^2^H_4_ (Sigma 741167) as isotope-labeled internal standards.) was added to each well of the frozen plate. The cells were then scraped, transferred to a new tube and dissolved by sonication followed by an incubation for 30 min at 1500 rpm at 4°C. The metabolite-containing supernatant was cleared by centrifugation at 20,000 × g for 10 min and subsequently transferred to a speedvac concentrator to fully evaporate the extraction buffer and stored at −80°C until further analysis. The remaining protein pellet from the extraction was used to determine protein concentration of the sample.

### Anion-Exchange Chromatography Mass Spectrometry (AEX-MS) for the analysis of anionic metabolites

Extracted metabolites were re-suspended in 150 μl of UPLC/MS grade water (Biosolve), of which 100 μl were transferred to p*olypropylene* autosampler vials (Chromatography Accessories Trott, Germany) before AEX-MS analysis. The samples were analysed using a Dionex ionchromatography system (Integrion Thermo Fisher Scientific) as described previously (61).

In brief, 5 μL of the resuspended polar metabolite extract were injected in push-partial mode, using an overfill factor of 1, onto a Dionex IonPac AS11-HC column (2 mm × 250 mm, 4 μm particle size, Thermo Fisher Scientific) equipped with a Dionex IonPac AG11-HC guard column (2 mm × 50 mm, 4 μm, Thermo Fisher Scientific). The column temperature was held at 30°C, while the auto sampler temperature was set to 6°C. A potassium hydroxide gradient was generated using a potassium hydroxide cartridge (Eluent Generator, Thermo Scientific), which was supplied with deionized water (Milli-Q IQ 7000, Millipore). The metabolite separation was carried at a flow rate of 380 μL/min, applying the following gradient conditions: 0–3 min, 10 mM KOH; 3–12 min, 10−50 mM KOH; 12–19 min, 50–100 mM KOH; 19–22 min, 100 mM KOH, 22–23 min, 100–10 mM KOH. The column was re-equilibrated at 10 mM for 3 min.

For the analysis of metabolic pool sizes the eluting compounds were detected in negative ion mode using full scan measurements in the mass range m/z 77 – 770 on a Q-Exactive HF high resolution MS (Thermo Fisher Scientific). The heated electrospray ionization (ESI) source settings of the mass spectrometer were: Spray voltage 3.2 kV, capillary temperature was set to 300°C, sheath gas flow 50 AU (arbitrary units), aux gas flow 20 AU at a temperature of 330°C and a sweep gas flow of 2 AU. The S-lens was set to a value of 60. The LC-MS data analysis was performed using the TraceFinder software (Version 5.1, Thermo Fisher Scientific). The identity of each compound was validated by authentic reference compounds, which were measured at the beginning and the end of the sequence. For data analysis the area of the deprotonated [M-H^+^]^−1^ or doubly deprotonated [M-^2^H]^−2^ isotopologue mass peaks of every required compound were extracted and integrated using a mass accuracy <3 ppm and a retention time (RT) tolerance of <0.05 min as compared to the independently measured reference compounds. If no independent ^12^C experiments were carried out, where the pool size is determined from the obtained peak area of the ^12^C monoisotopologue, the pool size determination was carried out by summing up the peak areas of all detectable isotopologues per compound. These areas were then normalized, as performed for un-traced ^12^C experiments, to the internal standards, which were added to the extraction buffer, followed by a normalization to the protein content or the cell number of the analyzed samples. The relative isotope distribution of each isotopologue was calculated from the proportion of the peak area of each isotopologue towards the sum of all detectable isotopologues. Natural abundance of the measured isotopologue was corrected using the AccuCor package (62).

### Sample preparation for folate detection

Cell extracts were prepared by homogenization of cell pellets by sonication in buffer containing 20 mM ammonia acetate, 0.1% ascorbic acid, 0.1% citric acid and 100 mM dithiothreitol at pH 7. Protein was removed by precipitation by addition of two volumes of acetonitrile and centrifugation (12,000 g at 4°C). Supernatants were transferred, lyophilized, stored at −80°C and re-suspended in dH_2_O before analysis. Folate analysis was carried out by ultraperformance liquid chromatography tandem mass spectrometry (UPLC-MS/MS) as described previously (63, 64). Lyophilized samples were resuspended in 25 uL water (milli-Q) and centrifuged for 5 min at 12,000 g at 4°C. Metabolites were resolved by reversed-phase UPLC (Acquity UPLC BEH C18 column, Waters Corporation, UK). Solvents for UPLC were as follows: Buffer A, 5% methanol, 95% Milli-Q water and 5 mM dimethylhexylamine at pH 8.0; Buffer B, 100% methanol, 5 mM dimethylhexylamine. The column was equilibrated with 95% Buffer A: 5% Buffer B. The sample injection volume was 20 uL. The UPLC protocol consisted of 95% Buffer A: 5% Buffer B for 1 min, followed by a gradient of 5–60% Buffer B over 9 min and then 100% Buffer B for 6 min before re-equilibration for 4 min. The metabolites were eluted at a flow rate of 500 μL/min and the wash step with 100% Buffer B was at flow rate of 600 μL/min. The UPLC was coupled to a XEVO-TQS mass spectrometer (Waters Corporation) operating in negative-ion mode using the following settings: capillary 2.5 kV, source temperature 150°C, desolvation temperature 600°C, cone gas flow rate 150 L/h, and desolvation gas flow rate 1200 L/h. Folates were measured by multiple reaction monitoring with optimized cone voltage and collision energy for precursor and product ions (63, 65). Peak areas were analysed by TargetLynx software (Waters Corporation, UK).

### Infection of mice with Toxoplasma

6–8 weeks old female C57BL/6 J mice were obtained from Charles River. All animal work was approved by local authorities (Landesamt für Natur, Umwelt und Verbraucherschutz Nordrhein-Westfalen, Germany LANUV 81–02.04.2020.A467) and animal procedures were carried out in accordance with European, national and institutional guidelines and according to good practice of animal handling. Mice were maintained at the Max Planck Institute for Biology of Ageing with 12 h light cycle and regular chow diet. When mice were 8–10 weeks old, following serial dilution in PBS, 50 RH*Δku80:mCherry* tachyzoites and 200 Me49:mScarlet tachyzoites were injected intraperitoneally. Tunicamycin (Sigma, #11089–65-9) was dissolved in 150 mM sucrose in PBS and injected intraperitoneally at 1 mg kg^−1^ as previously described (66, 67). ISRIB (Sigma, #1597403–47-8) was dissolved in DMSO at 6.25 mg ml^−1^ and subsequently diluted in PBS at 0.25 mg ml^−1^ and delivered intraperitoneally to mice at a dose of 2.5 mg kg^−1^ per day every other day in the morning as previously described in (68). For vehicle DMSO was diluted in PBS.

### PEC isolation

Euthanized C57BL/6 J mice (*n* = 5 per treatment) were injected with 6 ml cold 1× PBS using a 26-gauge needle. Mice were palpated for 1–2 min, following which fluid contents were aspirated out of the peritoneum. Content was spun down at 1,000 rpm for 5 min, washed, and resuspended in DMEM to a concentration of 2×10^6^ per ml. Samples were then pellet and snap frozen in liquid nitrogen until further downstream analysis was performed accordingly.

### Statistical analysis

All statistical analyses were performed using GraphPad Prism 9. A one-way ANOVA was used to compare means across multiple groups when testing an independent variable. A two ANOVA was used when testing the effects of two independent variables. Comparisons between two independent groups were performed using unpaired t-tests, and multiple unpaired t-tests were used when comparing several independent variables between two groups. To correct for multiple comparisons, the false discovery rate (FDR) was controlled using the two-stage step-up method of Benjamini, Krieger, and Yekutieli. The statistical test used for each analysis is specified in the figure legends.

### Newly created materials

Newly created cell lines, parasite lines, and plasmids available upon request.

## Supplementary Material

SupplementaryTableS1

SupplementaryTableS2

SupplementaryTableS3

ATF4metimmunitysupplementarymaterialr3

## Data Availability

The mass spectrometry proteomics data have been deposited to the ProteomeXchange Consortium via the PRIDE (60) partner repository with the dataset identifier PXD064338. All other data are available in the main text or the supplementary materials.
