# Supplementary material for: Mitochondria protect against an intracellular pathogen by restricting access to folate
Source: Science. Author manuscript; Available in PMC 2025 Sep 30. (PMC12483063; doi:10.1126/science.adr6326)
Supplement: ATF4metimmunitysupplementarymaterialr3 [file NIHMS2109554-supplement-ATF4metimmunitysupplementarymaterialr3.docx]

Supplementary Materials for

**Mitochondria protect against an intracellular pathogen by restricting access to folate**

Tânia Catarina Medeiros^1^, Jana Ovciarikova^2^, Xianhe Li^1^, Patrick Krueger^1^, Tim Bartsch^3^, Silvia Reato^3,4^, John C. Crow^3^, Michelle Tellez Sutterlin^1^, Bruna Martins Garcia^1^, Irina Rais^1^, Kira Allmeroth^5^, Matías D. Hartman^5^, Martin S. Denzel^5^, Martin Purrio^6^, Andrea Mesaros^6^, Kit-Yi Leung^7^, Nicholas D.E. Greene^7^, Lilach Sheiner^2^, Patrick Giavalisco^8^, and Lena Pernas^1,3,4*^

**Affiliations:**

^1^Metabolism of Infection Group, Max Planck Institute for Biology of Ageing, Cologne, Germany

^2^ **Glasgow Centre for Parasitology, University of Glasgow, Scotland, UK**

^3^ **Dept. Microbiology, Immunology & Molecular Genetics, University of California Los Angeles, USA**

^4^Howard Hughes Medical Institute, Chevy Chase, MD USA

^5^Metabolic and Genetic Regulation of Ageing, Max Planck Institute for Biology of Ageing, Cologne, Germany

^6^Phenotyping Core Facility, Max Planck Institute for Biology of Ageing, Cologne, Germany

^7^Developmental Biology & Cancer Department, UCL, Great Ormond Street Institute of Child Health, University College London, UK

^8^Metabolomics Core Facility, Max Planck Institute for Biology of Ageing, Cologne, Germany

*Correspondence should be addressed to: lfpernas@mednet.ucla.edu

**This PDF file includes:**

Figs. S1 to S16

**Other Supplementary Materials for this manuscript include the following:**

Table S1. Protein abundance in uninfected and *Toxoplasma-*infected cells
 Table S2. Nanostring analysis of genes induced by *Toxoplasma* and tunicamycin
 Table S3. List of primers used


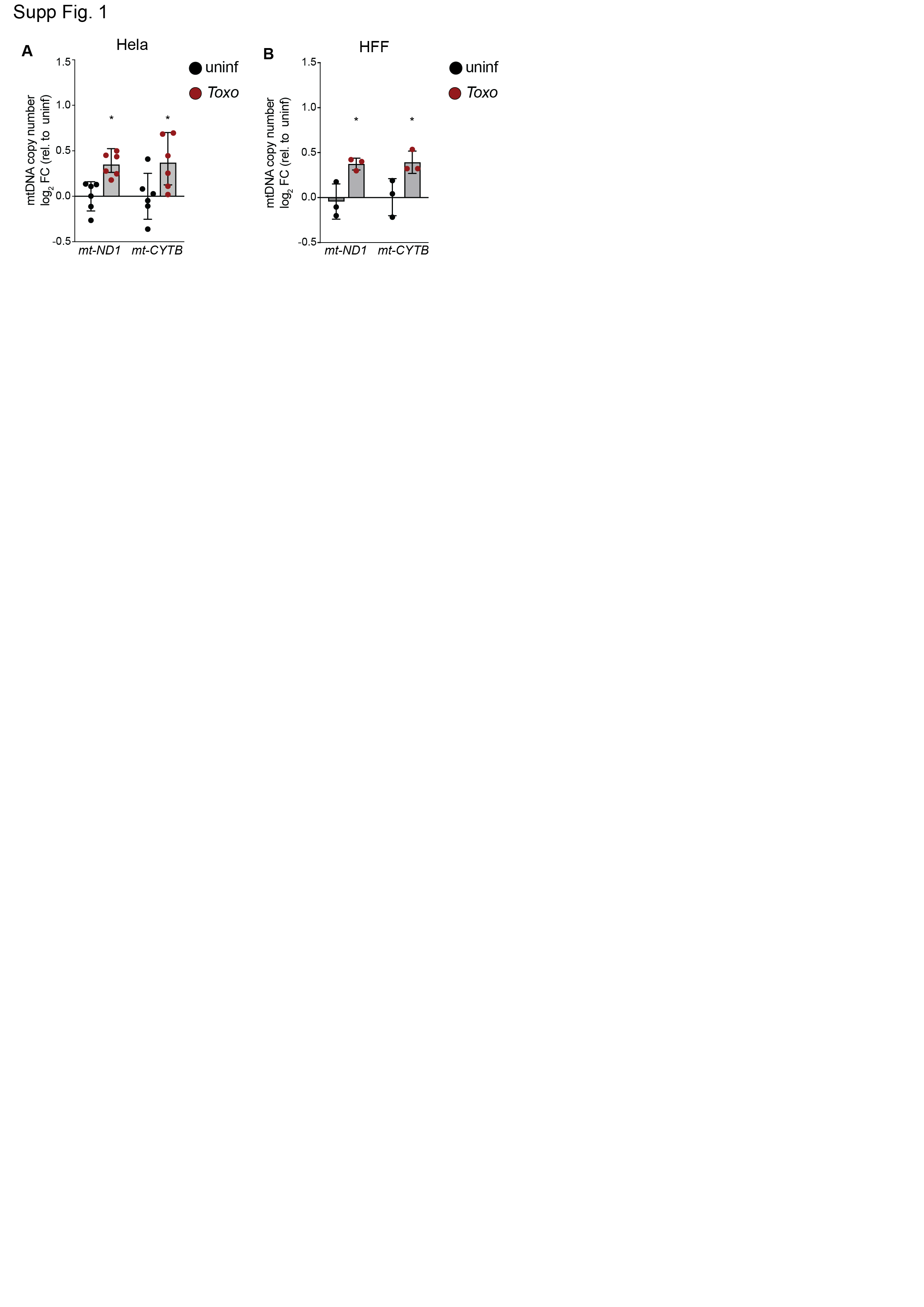


Fig. S1. *Toxoplasma* infection drives an increase in mtDNA copy number in both cancer and primary cell lines. mtDNA levels assessed by qPCR for *mt-ND1* and *mt-CYTB* (normalized to *RUNX2*) in (A) HeLa cells and (B) primary human foreskin fibroblasts (HFFs) that were uninfected or infected with *Toxoplasma* at a multiplicity of infection (MOI) of 4 and harvested at 24 hours post infection (hpi). Data are mean ± SD of (A) n=6 independent cultures and (B) n=3 independent cultures; *p<0.05; for uninfected versus infected by means of t-tests analysis.


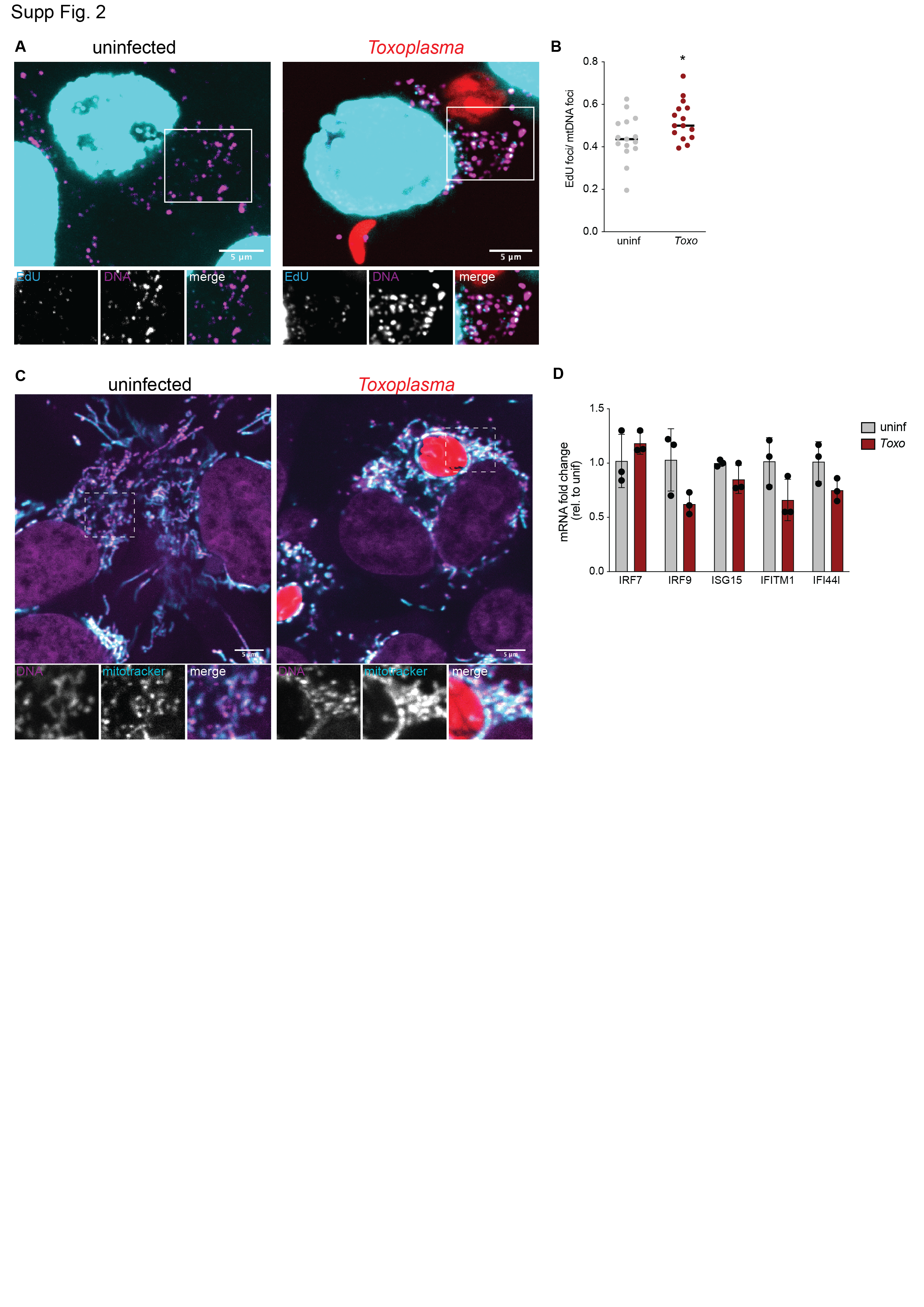
 Fig. S2. *Toxoplasma* infection increases intramitochondrial DNA levels
(A) Representative immunofluorescence images of uninfected and *Toxoplasma-*mCherry-infected ES-2 cells following 50 uM EdU labeling for 1h and fixed at 16 hpi. EDU: 5-ethynyl-2-deoxyuridine. (B) Quantification of EdU puncta per mtDNA (DNA+) foci in n=15 cells as in (A); *p<0.05 by unpaired t-test analysis. (C) Representative live-cell images of uninfected and *Toxoplasma-*mCherry-infected ES-2 cells labeled with MitoTracker Deep Red (mitotracker) and the DNA dye picogreen (DNA) and imaged at 24 hpi. Scale bar, 5 µm. (D) Uninfected and *Toxoplasma*-infected cells (MOI=4) were analyzed by qPCR at 24 hpi for the indicated transcripts. Transcripts were normalized to *ACTB* and are relative to uninfected samples. Data are mean ± SD of n=3 independent cultures.

**
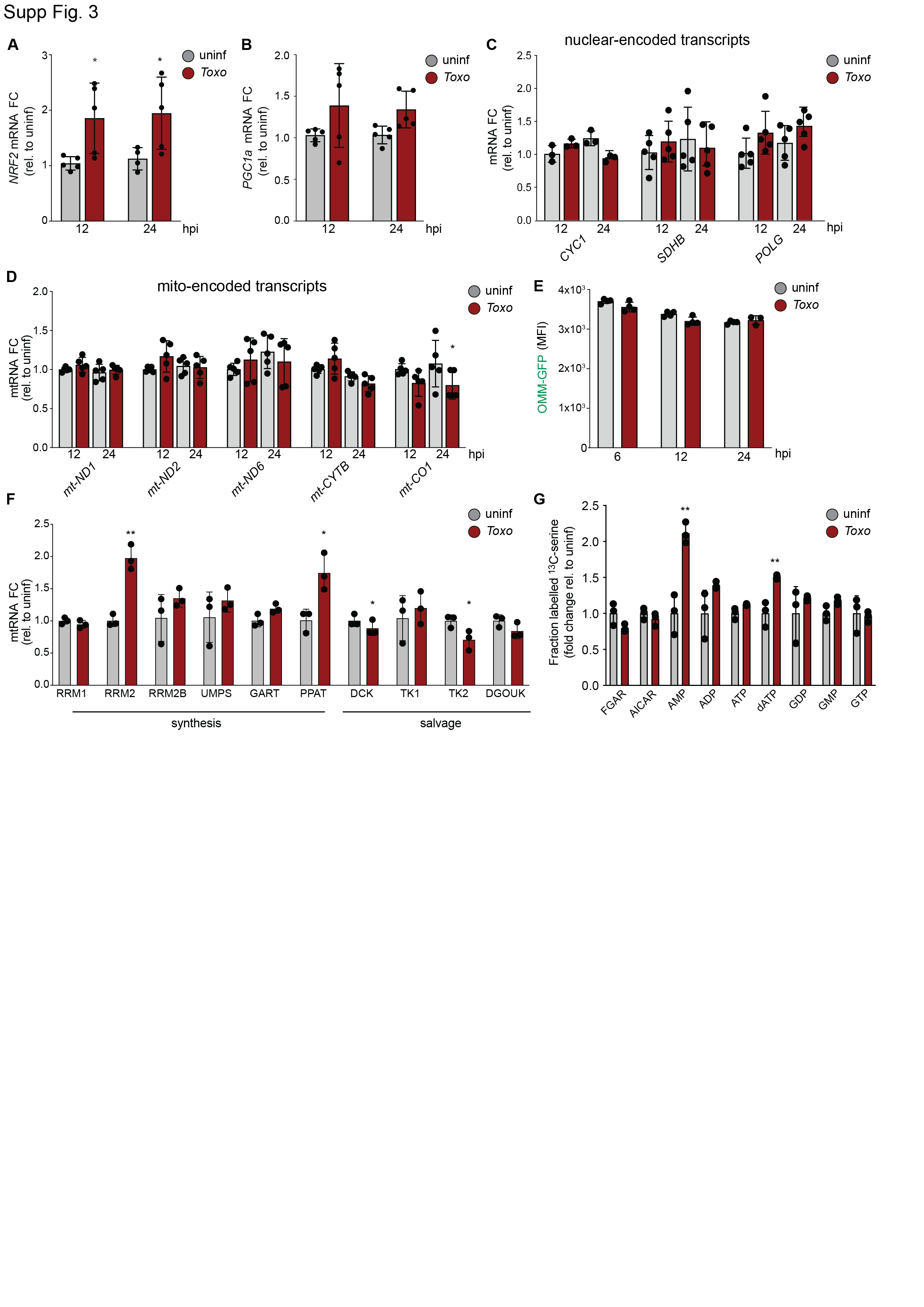
**

**Fig. S3. *Toxoplasma* infection does not induce mitochondrial biogenesis.** Uninfected and *Toxoplasma*-infected cells (MOI=4) were analyzed by qPCR at the indicated timepoints for (**A**) *NRF2*; (**B**) PGC1α; (**C**) the nuclear-encoded mitochondrial transcripts *CYC1*, *SDHB*, *POLG;* and (**D**) the mitochondrial-encoded transcripts *mt-ND1, mt-ND2, mt-ND6, mt-CYTB and mt-CO1.* Transcripts were normalized to *ACTB* and are relative to uninfected samples. Data are mean ± SD of n=5 independent cultures, *p<0.05; for uninfected versus infected by means of two-way ANOVA analysis. (**E**) GFP fluorescence intensity (MFI) determined by flow cytometry analysis of cells stably expressing GFP targeted to the outer mitochondrial membrane (OMM) in uninfected cells or cells infected with RH-mCherry expressing *Toxoplasma* at 6, 12 and 24 hpi. Data are mean ± SEM of n=3 independent cultures. ES-2 cells used for all experiments. (**F**) Uninfected and *Toxoplasma*-infected cells (MOI=4) were analyzed by qPCR at 24 hpi for the indicated transcripts; transcripts were normalized to *ACTB* and are relative to uninfected samples. *p<0.05; **p<0.01; for uninfected versus infected by means of unpaired t-test analysis. (**G**) WT ES-2 cells were infected with *Toxoplasma,* and at14 hpi media was replaced with 0.2 mM L-serine^13^C-containing media for 10 h. Cells were harvested 24 hpi and analyzed for fraction enrichment of serine-derived ^13^C nucleotides. Data are ± SD of n=3 independent cultures; **p<0.01; for uninfected versus infected by means of multiple unpaired t-test analysis.

**
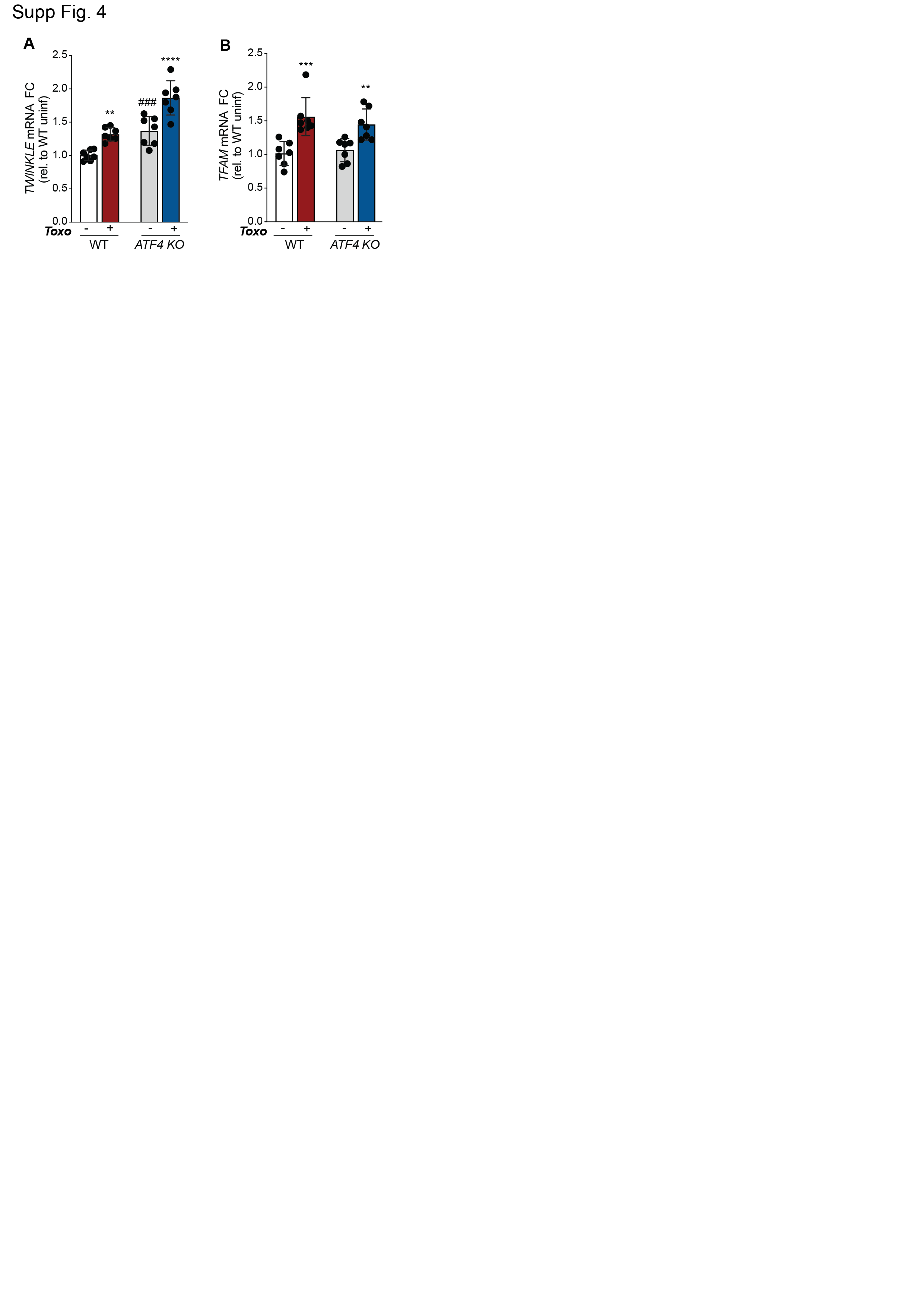
Fig. S4. *TWNK* and *TFAM* are induced independently of ATF4 during *Toxoplasma* infection.** Uninfected or *Toxo-*infected WT and *ATF4* KO ES-2 cells were analyzed by qPCR for (**A**) *TWINKLE* and (**B**) *TFAM* at 24 hpi. (**A** and **B**) Transcript levels were normalized to *ACTB* and are relative to WT uninfected. Data are mean ± SD of n=7 independent cultures. **p < 0.01; ***p<0.001; ****p<0.001 for uninfected versus *Toxo*-infected, ###*p* < 0.001 for WT versus *ATF4 KO* by means of two-way ANOVA analysis.


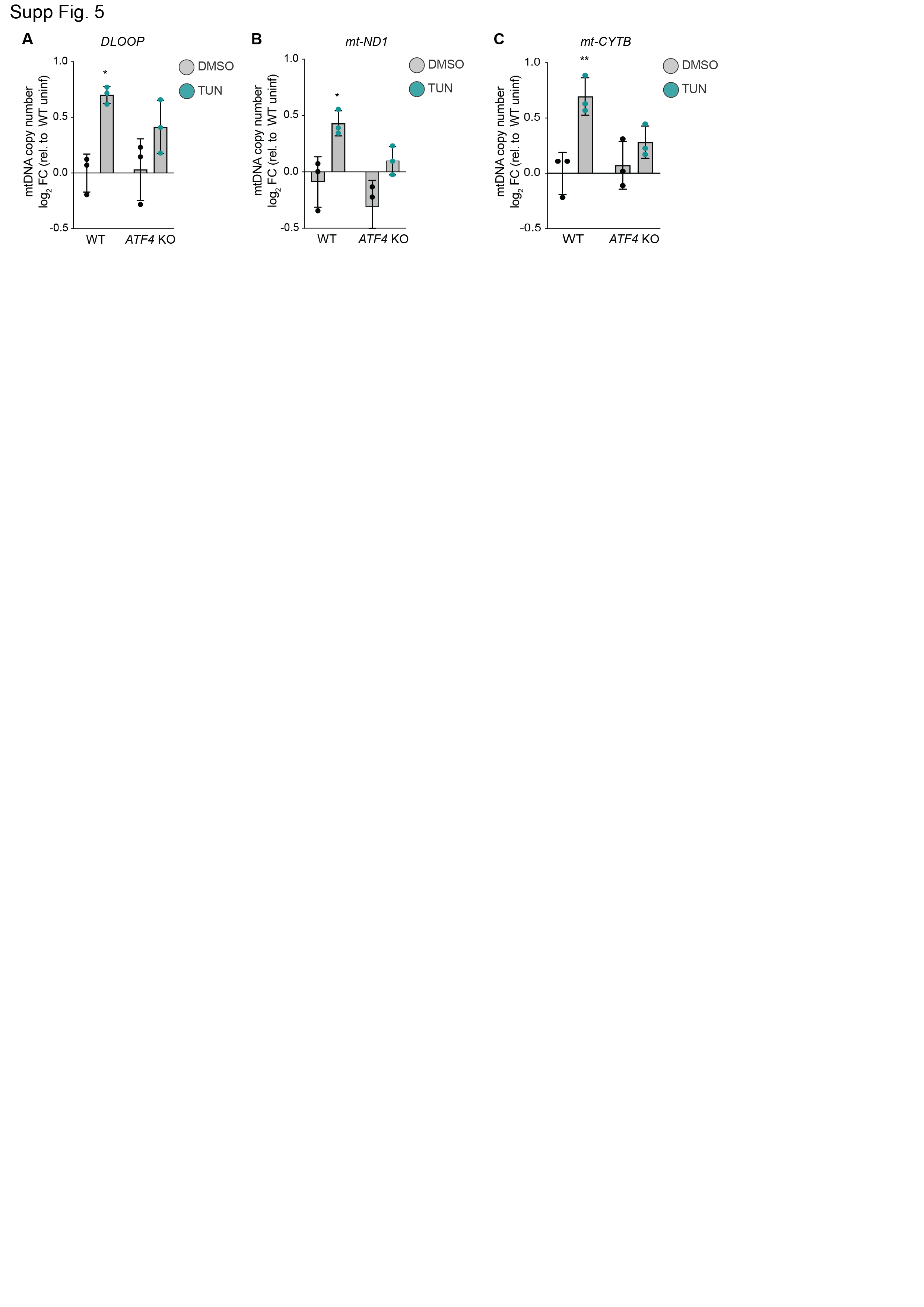


**Fig. S5. ISR activation increases mtDNA levels.** mtDNA levels were assessed by qPCR of (**A**) *DLOOP,* (**B**) *mt-ND1* and (**C**) *mt-CYTB* (normalized to *RUNX2*) in WT and *ATF4* KO ES-2 cells that were untreated or treated with tunicamycin (3 μg/ml) for 16 hours. Data are mean ± SD of n=3 independent cultures; *p<0.05; **p<0.001; for untreated versus tunicamycin-treated by means of two-way ANOVA analysis.


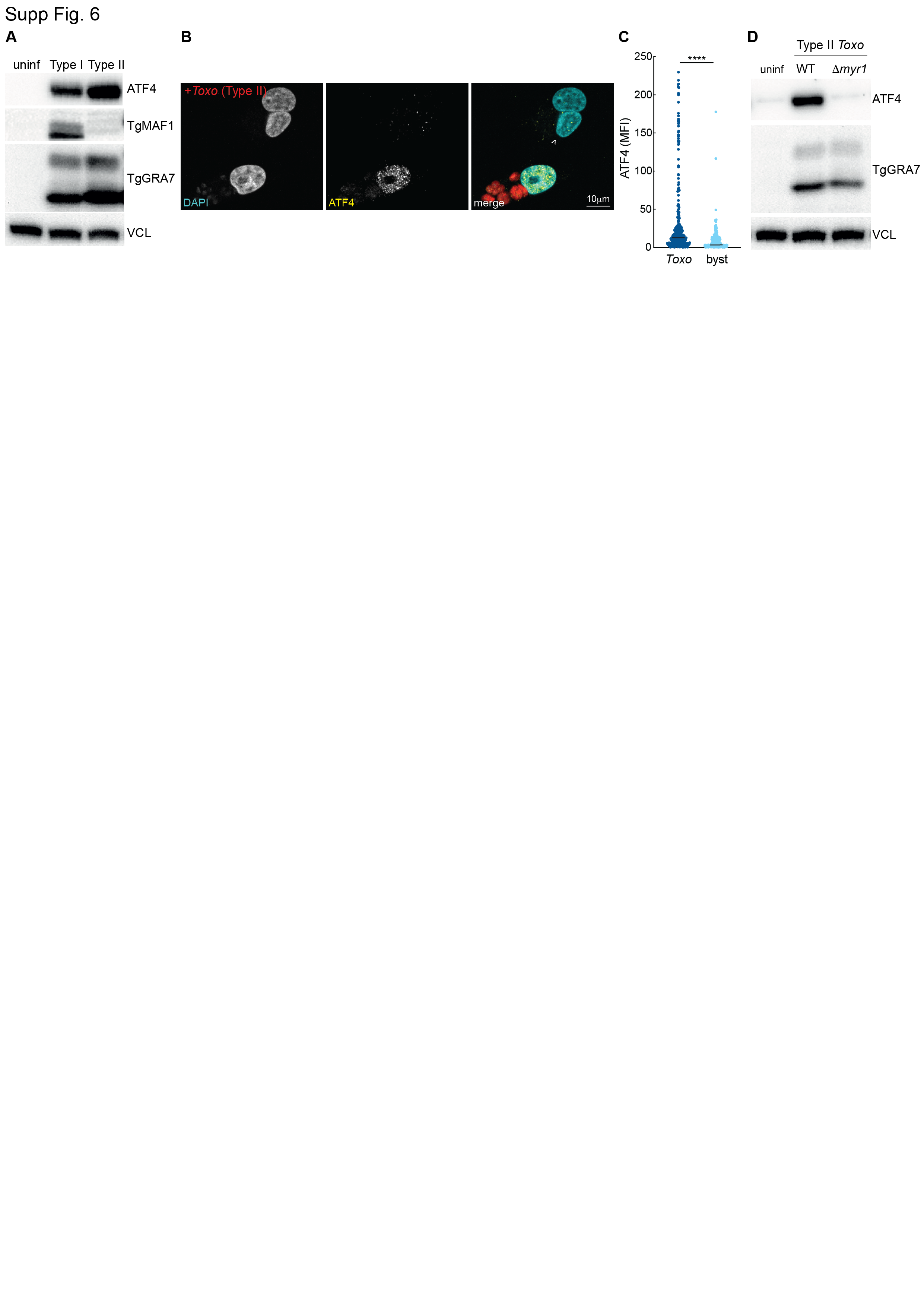


**Fig. S6. Type II *Toxoplasma* parasites drive ATF4 activation in a manner similar to Type I *Toxoplasma* parasites.** (**A**) Immunoblot (IB) analysis of lysates from ES-2 cells that were uninfected (uninf) or infected with Type I and Type II *Toxoplasma* parasites at an MOI=4 at 24 hpi; ATF4, ~50 kDa; Vinculin (VCL), ~124 kDa; *Toxoplasma* mitochondrial association factor (TgMAF1) ~60 kDa, and *Toxoplasma* GRA7 (TgGRA7), ~27 kDa. (**B**) Immunofluorescence images of uninf cells and cells infected with mScarlet-expressing Type II *Toxoplasma* at 24 hpi; scale bar, 10 μm. (**C**) Quantification of mean fluorescence intensity (MFI) of ATF4 in the nuclei of cells in images as in (B); byst: bystander cell. (**D**) IB analysis of lysates from ES-2 cells that were uninfected (uninf) or infected with indicated *Toxoplasma* parasites at an MOI=4 at 24 hpi.

**
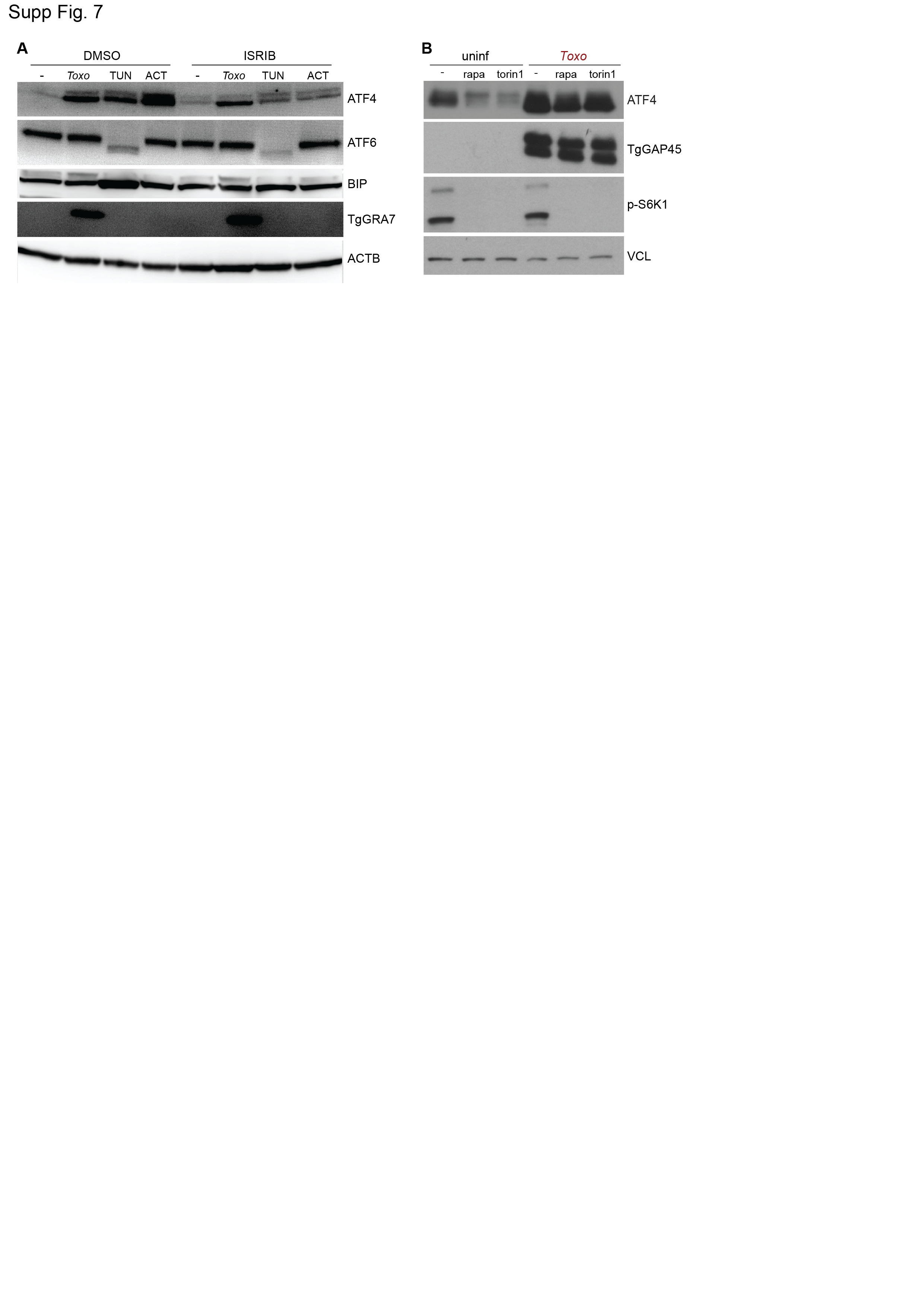
**

**Fig. S7. ATF4 activation during infection is mTOR independent.** (**A**) Immunoblot (IB) analysis of lysates from ES-2 cells that were treated as indicated: uninfected (uninf); *Toxoplasma (Toxo)* at MOI=4; tunicamycin (TUN; 3 μg/ml); or actinonin (ACT; 50 μM) with or without ISRIB (200 nM) for 24h: ATF6, ~84kDa; BiP, ~78 kDa); ATF4, ~50 kDa; α-Actin (ACTB), ~35 kDa) and *Toxoplasma* GRA7 (TgGRA7), ~27 kDa. (**B**) Immunoblot analyses of lysates from uninfected and *Toxo-*infected (MOI: 4) ES-2 cells at 24 hpi and co-treated with DMSO, rapamycin (rapa; 20 nM) or torin1 (250 nM) and probed with the following antibodies: ATF4; Phospho-p70 S6 Kinase Thr389 (p-S6K1), ~70 and 80 kDa; Vinculin (VCL), ~124 kDa; and *Toxoplasma* GAP45 (TgGAP45,) ~45 kDa.


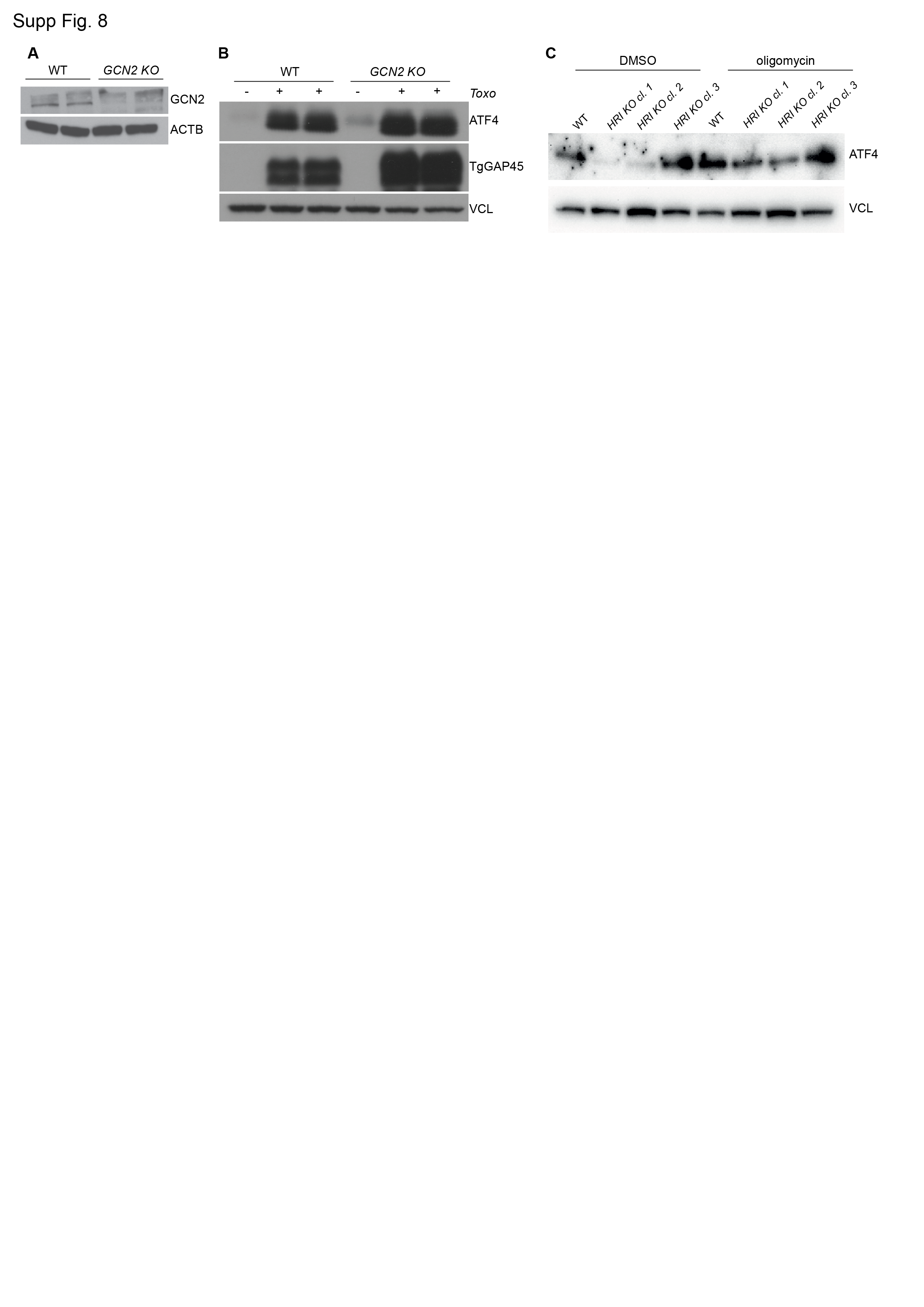


**Fig. S8. Role of ISR kinases in ATF4 activation during infection.** (**A**) Immunoblot (IB) analysis of lysates from WT and GCN2 KO ES-2 cells. GCN2 ~220 kDa, ACTB, ~45kD. (**B**) IB analyses of WT and GCN2 KO cells that were uninfected or infected at MOI=4 and harvested at 24 hpi. ATF4, ~50 kDa; Vinculin (VCL), ~124 kDa; and *Toxoplasma* GAP45 (TgGAP45,) ~45 kDa. (**C**) IB analyses of lysates from WT and HRI KO clones following 24 h treatment with oligomycin (2μM).


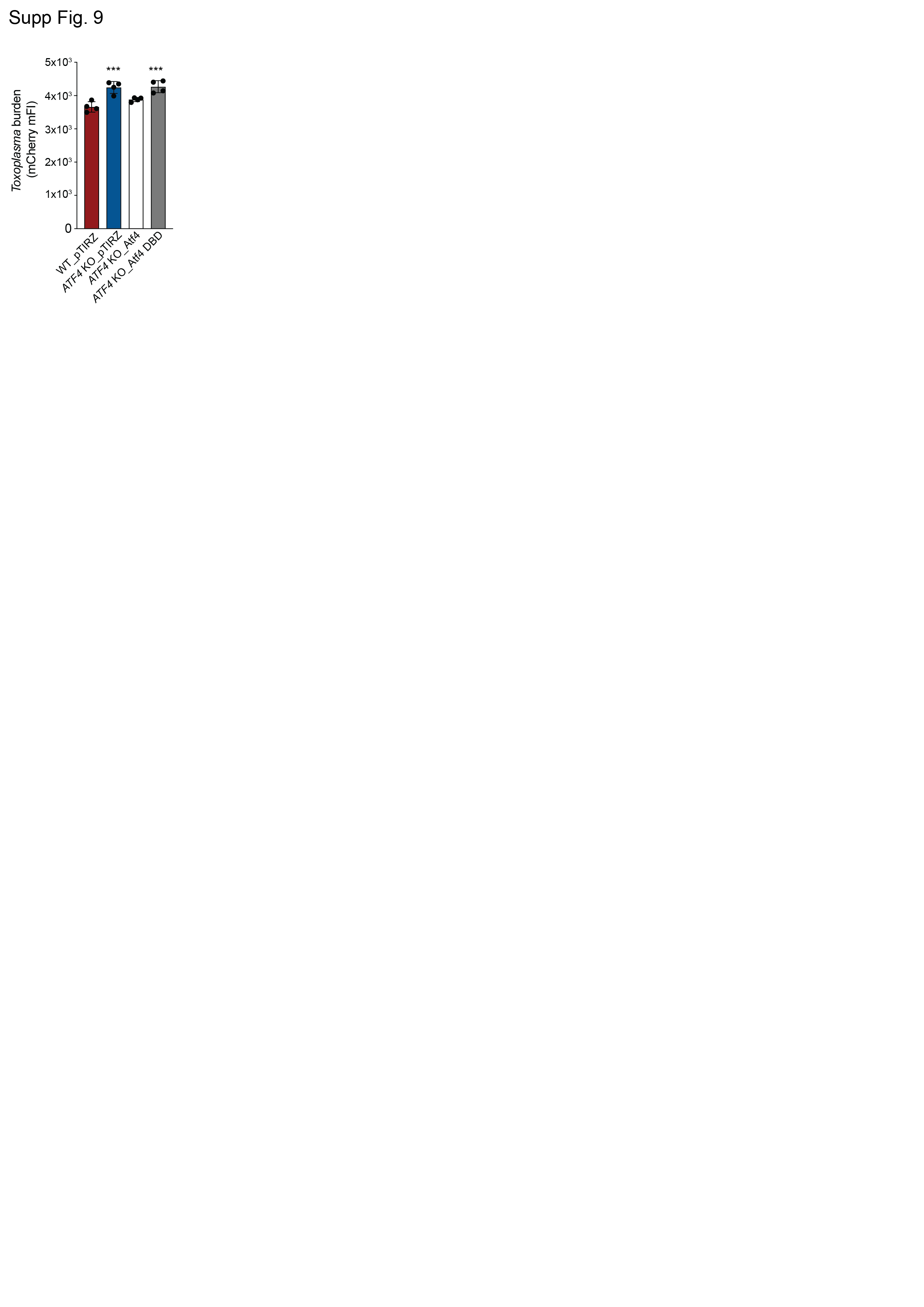


**Fig. S9. ATF4 requires its DNA-binding domain to restrict *Toxoplasma* growth.** (**G**) WT and ATF4 KO ES-2 cells expressing the indicated constructs were infected with *Toxoplasma*  and analyzed 24 hpi by means of flow cytometry for *Toxoplasma* burden (mCherry median FI). Data are mean ± SEM of three biological experiments, ***p < 0.001 by means of one-way ANOVA analysis.

**
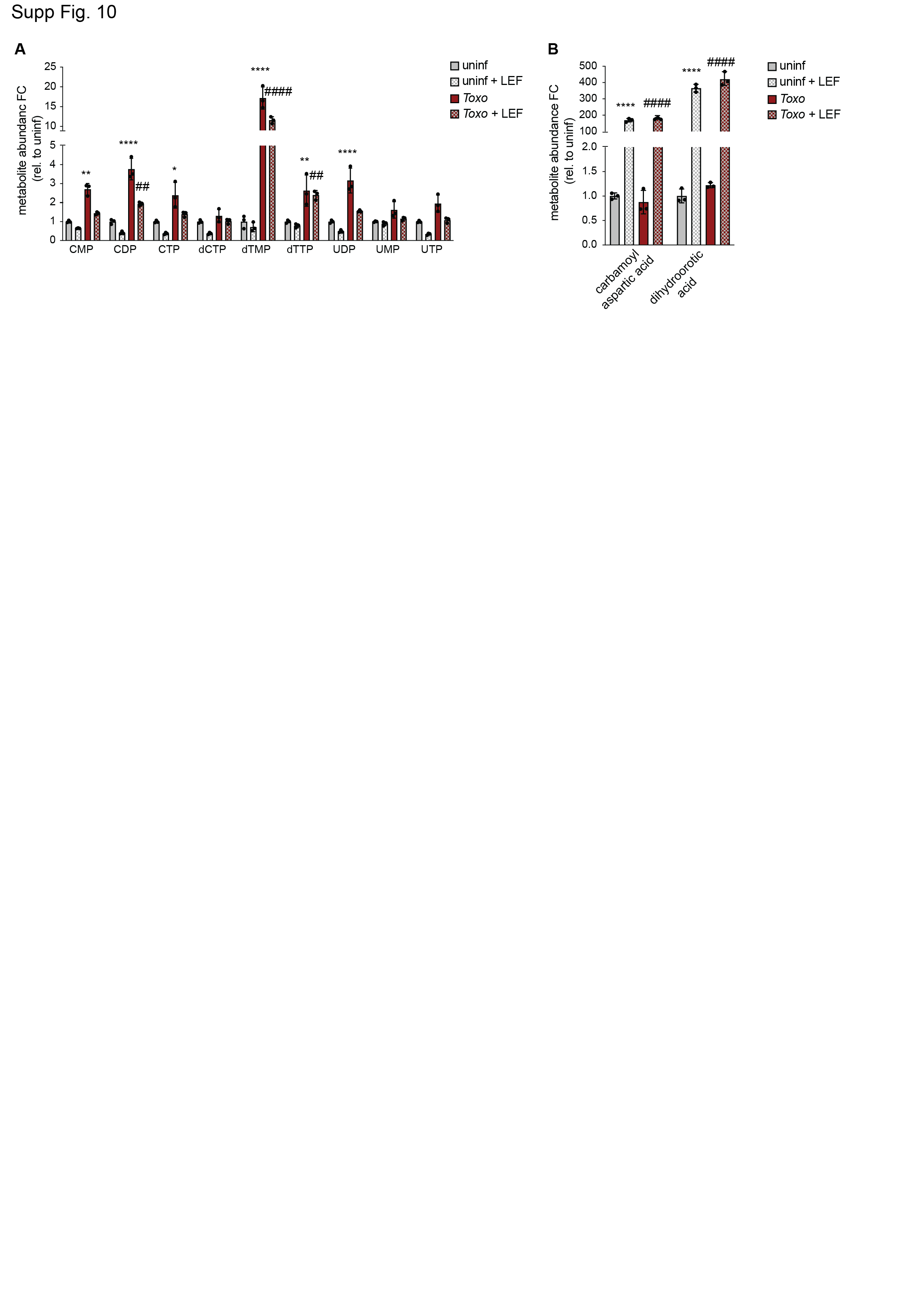
Fig. S10. dTMP levels are increased in *Toxoplasma-*infected cells during host pyrimidine synthesis inhibition.** (**A**) Abundance of total pyrimidine intermediates metabolites in ES-2 uninfected and *Toxoplasma*-infected cells at MOI=4 and treated with DMSO or leflunomide (LEF) (50 μM). Data are mean ±SEM of n=3 independent cultures, and are normalized to cell number, **p<0.01; ****p<0.0001 for uninf versus infected and ##p<0.01; ####p < 0.0001 for DMSO versus LEF treatment by means of two-way ANOVA analysis. (**B**) Total abundance of carbamoyl aspartic acid and dihydroorotic acid in samples treated as in (A). Data are mean ±SEM of n=3 independent cultures, and are normalized by cell number, ****p<0.0001 for uninfected versus infected and ####p < 0.0001 for DMSO versus LEF treatment by means of two-way ANOVA analysis.


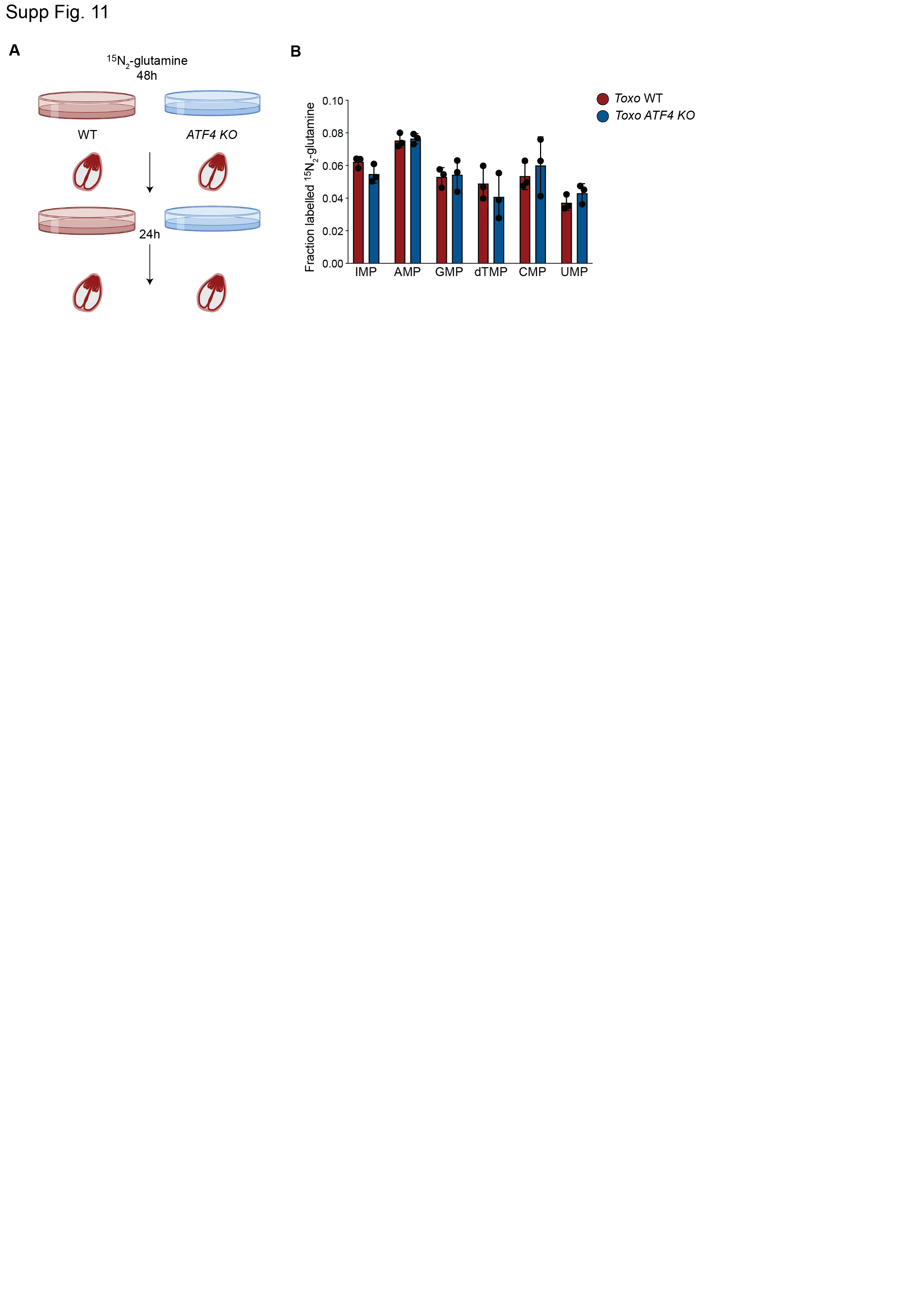
Fig. S11. Loss of ATF4 does not promote nucleotide acquisition by *Toxoplasma*. (A) Workflow for assessing *Toxoplasma* nucleotide acquisition from host cells. WT and *ATF4* KO ES-2 cells were culture with 2 mM L-glutamine^15^N_2_ for 48 h. Cells were then infected with *Toxoplasma* in the absence of the isotopologue L-glutamine^15^N_2_. After 24h the *Toxoplasma*-enriched fraction was pelleted for metabolite extraction. (B) Fraction enrichment of glutamine-derived ^15^N nucleotides of *Toxoplasma*-purified extracts from WT and *ATF4* KO cells as described in (A). Data are ± SD of n=3 independent cultures.


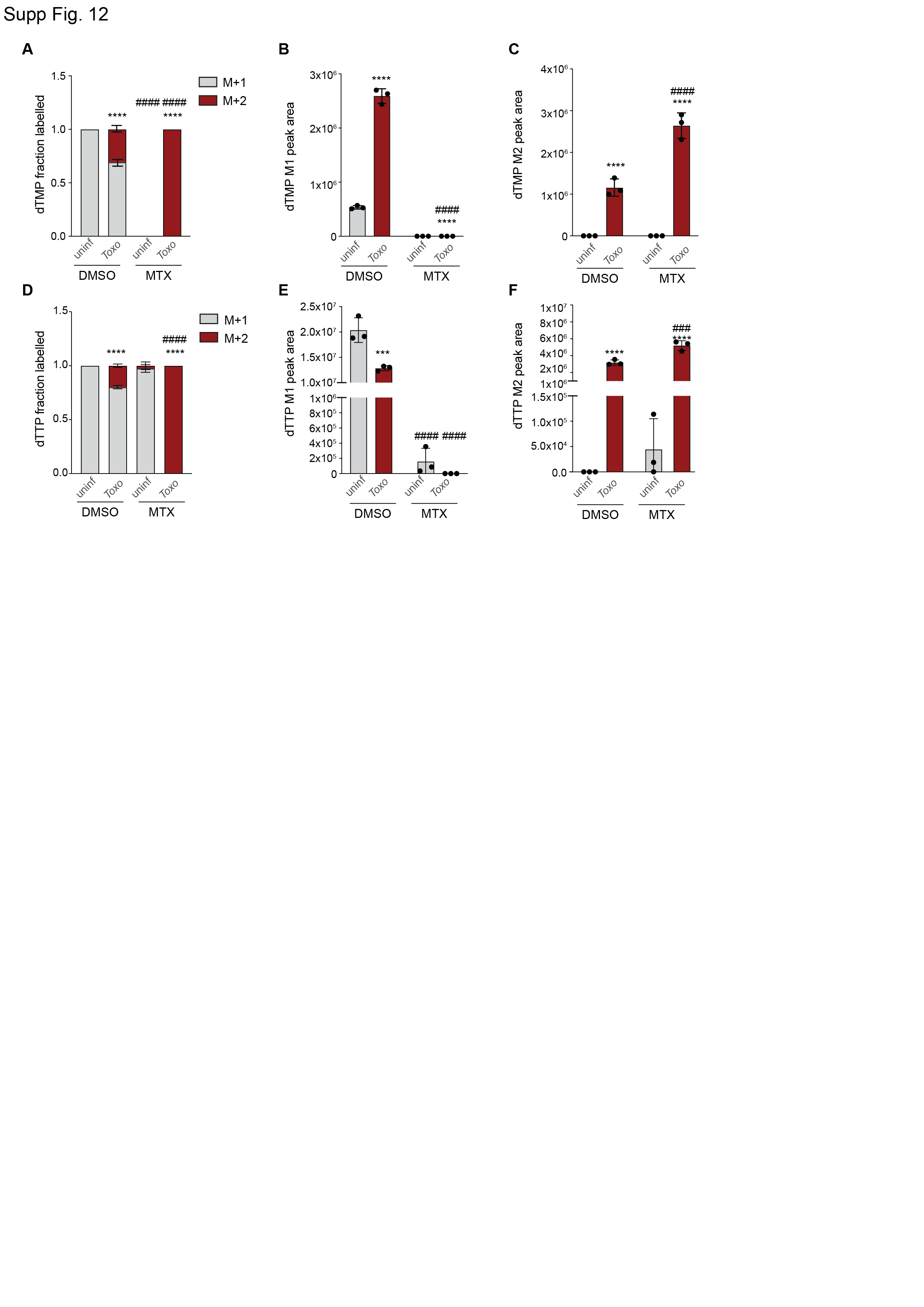
Fig. S12. Host 1C-metabolism competes with *Toxoplasma* dTTP synthesis (A) WT ES-2 cells cultured for 24 h with [2,3,3-^2^H] serine-containing media and either uninfected or infected with mCherry-expressing *Toxoplasma* ± methotrexate (MTX, 200nM). Cells were then analyzed for the dTMP fraction labeled and peak area of (B) M+1 dTMP and (C) M+2 dTMP. (D) dTTP fraction labeled and peak area of (E) M+1 dTMP and (F) M+2 dTMP. Data are mean ± SD of n=3 independent cultures, ***p<0.001, ****p<0.0001 for uninfected versus infected and ####p<0.0001 for DMSO versus MTX treatment by means of two-way ANOVA analysis.

**
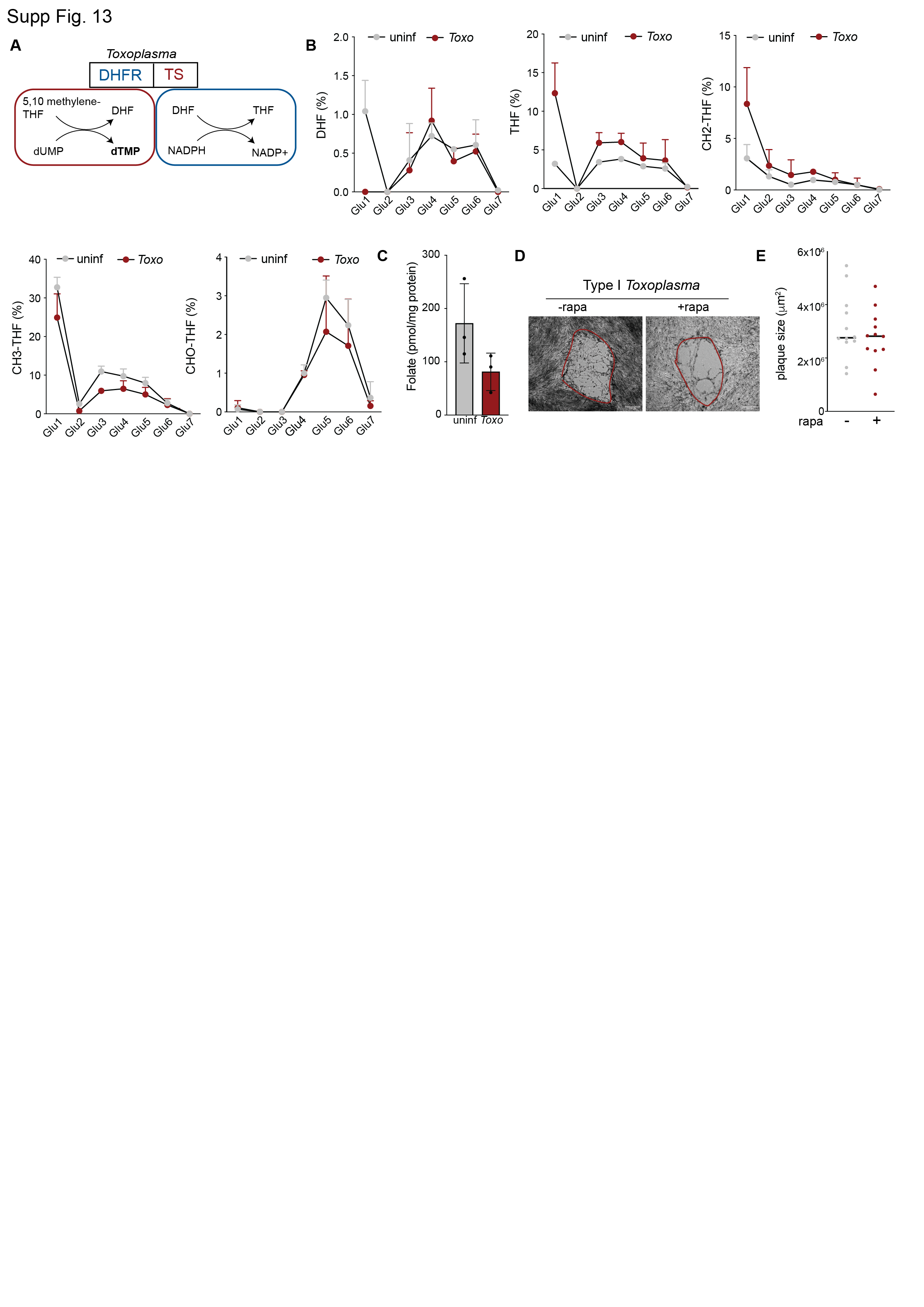
Fig. S13.** **Folate levels and *Toxoplasma* infection.** (**A**) Schematic of bifunctional dihydrofolate reductase thymidylate synthase (DHFR-TS) of *Toxoplasma gondii*. (**B**) Glutamylated folate species and (**C**) total folates of uninfected and infected cells analyzed in Fig. 3F. (**D**) Plaque assays of *Toxoplasma* Type I parasites (control for Di-Cre parasites in Fig. 3K, L) ± rapa (50nM) in human foreskin fibroblasts at 7 dpi; scale bar: 500 μM (**E**) Quantification of plaques as in (**D**), n=12.


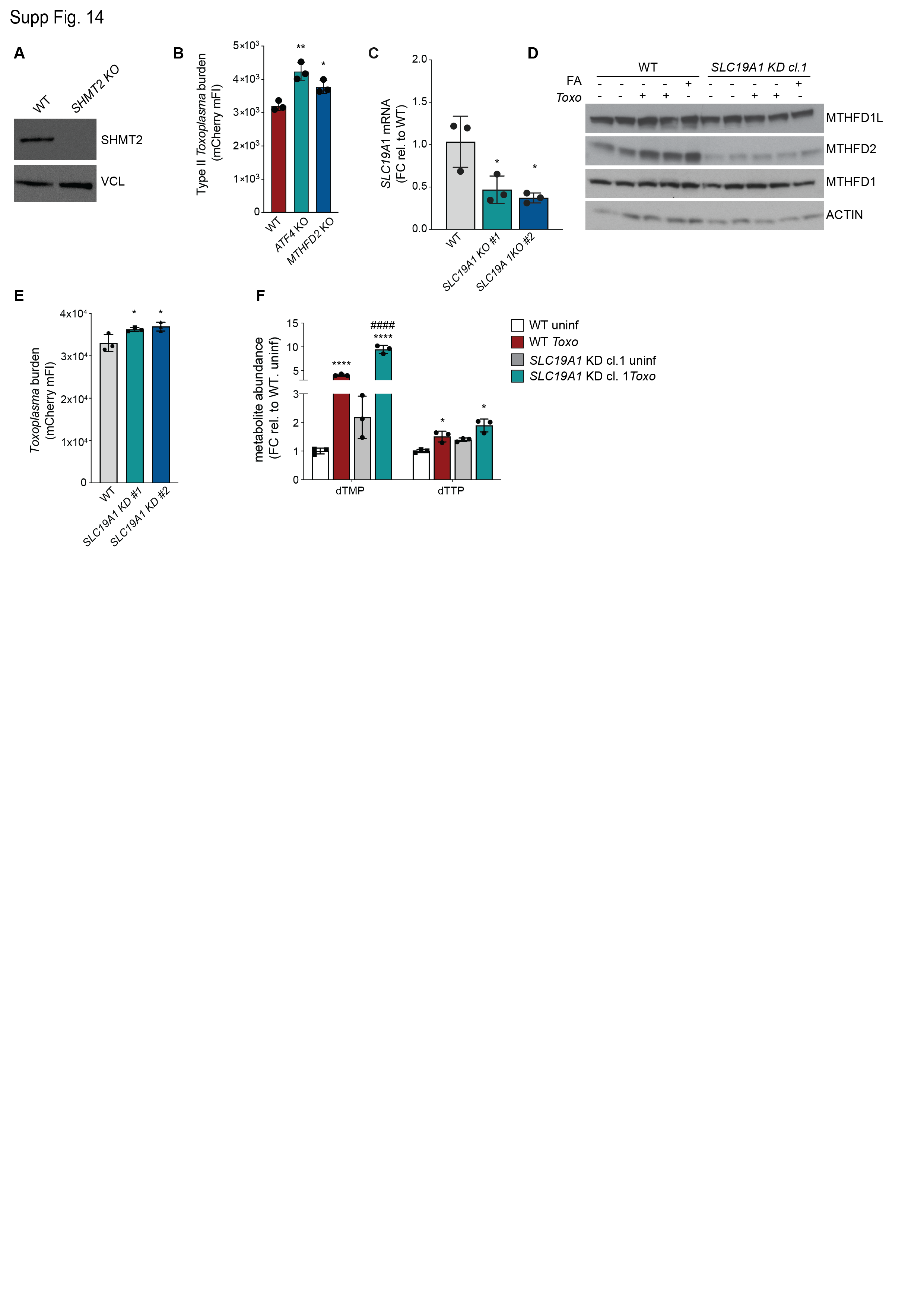


**Fig. S14. Increased parasite proliferation and dTMP levels in a model of mitochondrial 1C-dysfunction.** (**A**) IB of WT and SHMT2 KO ES-2 cells vinculin (VCL), ~124 kDa. (**C**) WT and SLC19A KO cells were analyzed by qPCR for *SLC19A1*. Transcripts were normalized to *ACTB*. Data are mean ± SD of n=3 independent cultures. (**B**) WT, ATF4 KO, and MTHFD2 KO ES-2 cells were infected with Type II mScarlet-expressing *Toxoplasma* and analyzed 48 hpi by means of flow cytometry for *Toxoplasma* burden (mScarlet median FI). Data are mean ± SEM of three biological experiments. *p < 0.05; **p < 0.01 by means of one-way ANOVA analysis. (**C**) WT and SLC19A KO cells were analyzed by qPCR for *SLC19A1*. Transcripts were normalized to *ACTB*. Data are mean ± SD of n=3 independent cultures. *p<0.05 by means of one-way ANOVA analysis. (**D**) WT and *SLC19A1* knockdown (KD) ES-2 cells were cultured for 4 days in RPMI medium lacking folic acid and uninfected, infected with *Toxoplasma* (MOI: 4) cells or supplemented with folic acid (2 μM). After 24h, samples were analyzed by immunoblotting for the indicated antibodies MTHFD1L, ~106 kDa; MTHFD2, ~32 kDa; MTHFD1, ~40 kDa and α-Actin (ACTB), ~35 kDa. (**E**) WT and S*LC19A1* KD ES-2 cells were infected with *Toxoplasma* and analyzed 24 hpi by means of flow cytometry for *Toxoplasma* burden (mCherry median FI). Data are mean ± SEM of three biological experiments, *p < 0.05 for WT versus *SLC19A1* KO, by means of one-way ANOVA analysis. (**F**) Total abundance of dTMP and dTTP of WT and S*LC19A1* KO ES-2 uninfected and *Toxo*-infected cells. Data are mean ± of SED n=3 independent cultures, and normalized by cell number, *p<0.05; ****p<0.0001 for uninfected versus infected cells and ####p < 0.0001 for WT versus *SLC19A1* KO cells by means of two-way ANOVA analysis.


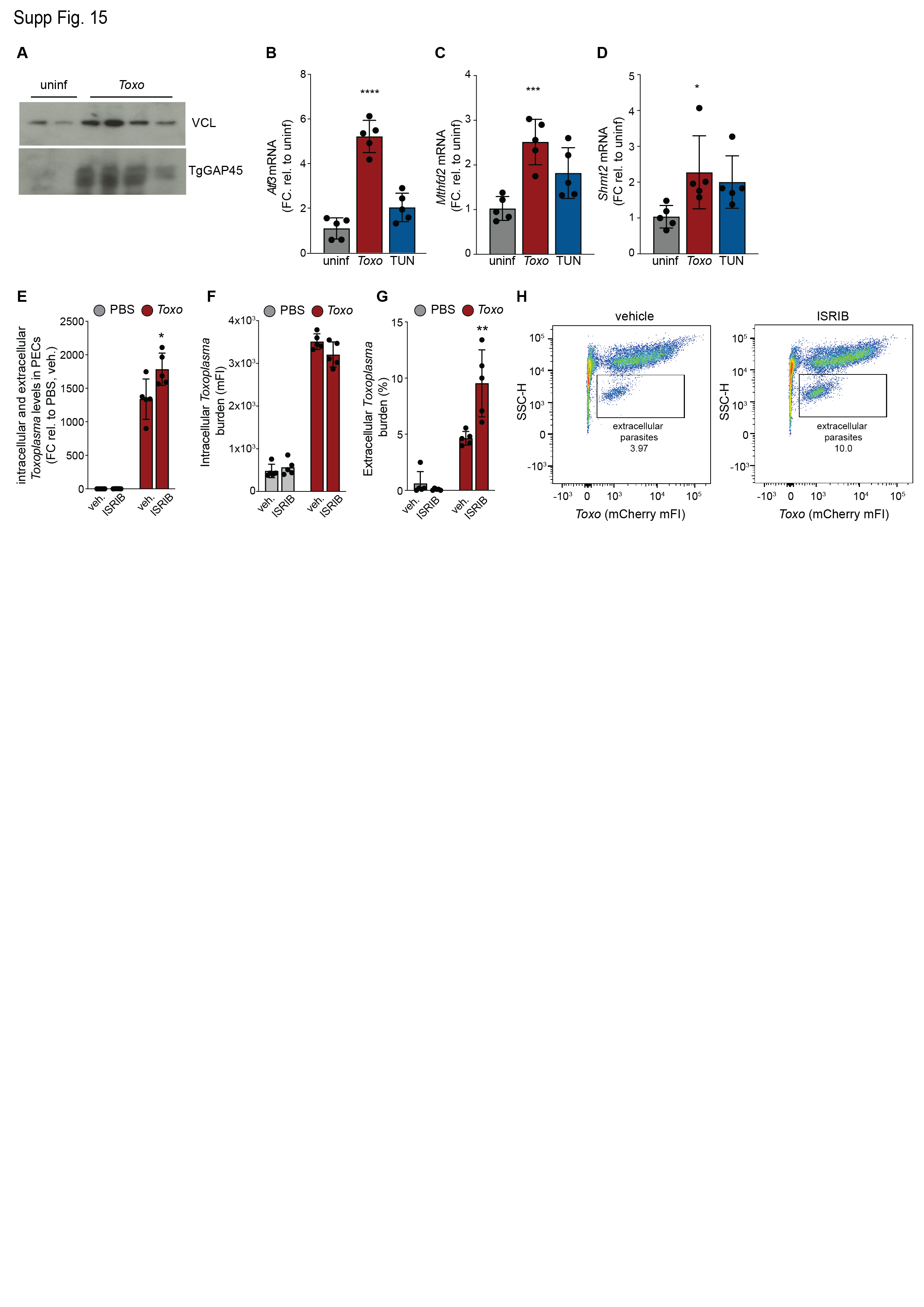


**Fig. S15. *Toxoplasma* infection and tunicamycin treatment induce ATF4 targets in vivo.** (**A**) Immunoblot (IB) analysis of lysates of peritoneal exudate cells (PECs) isolated at 5 dpi from mice that there uninfected or infected with 50 tachyzoites: *Toxoplasma* GAP45 (TgGAP45), ~45 kDa; Vinculin (VCL), ~124 kDa; (**B**-**D**) Mice were injected intraperitoneally with 1x PBS or 50 tachyzoites in 1x PBS or with tunicamycin (1 mg kg^−1^ body weight). At 5 days post injection (dpi) peritoneal exudate cells (PECs) were isolated and analyzed for the indicated ATF4 targets genes by qPCR. Transcripts are normalized to *Hprt* and relative to untreated uninfected PECs mice (n=5). * p<0.05; *** p<0.001; and ****p<0.0001 by means of one-way ANOVA analysis. (**E**) Peritoneal exudate was isolated 5 dpi from mice treated as in (**A**) and analyzed by qPCR for *Toxoplasma* *SAG1* levels. DNA levels were normalized to *Hprt.* Data are mean ± SD of n=5 mice, *p<0.05 by unpaired t-tests. Peritoneal exudate isolated from mice treated as in (**A**) was analyzed by means of flow cytometry for (**F**) intracellular *Toxoplasma* parasite burden (mCherry median FI) and (**G**) percentage of extracellular parasites. Data are mean ± SD of n=5 mice, *p<0.05; **p<0.01 by unpaired t-test analysis (vehicle vs. ISRIB). (**H**) Representative scatter plots of peritoneal exudate isolated from mice treated as in (**A**).

**
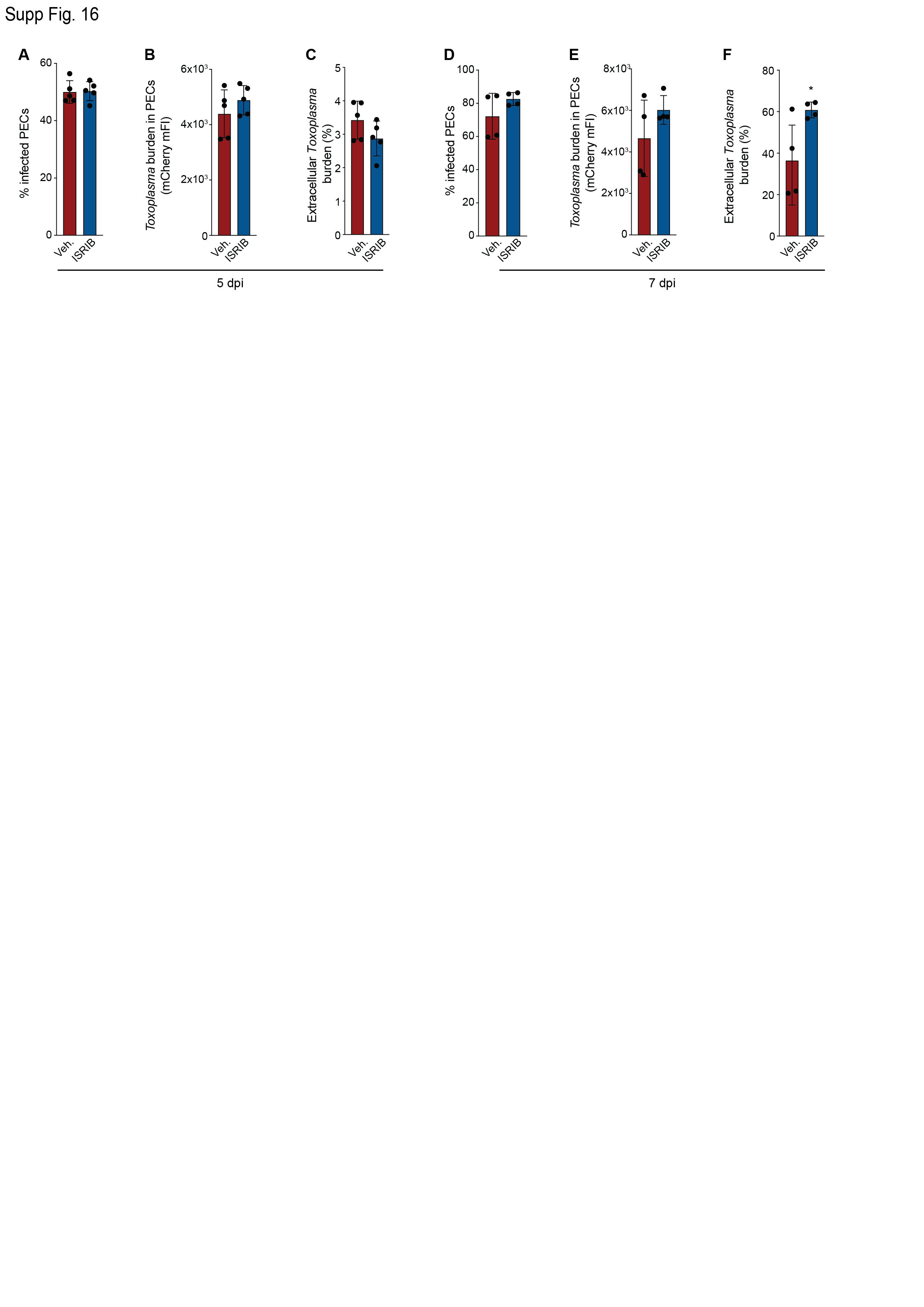
Fig. S16. Mice treated with ISRIB during infection have increased peritoneal parasitemia.** (**A**) Mice were injected intraperitoneally with 1x PBS or 200 mScarlet-expressing Type II tachyzoites in 1x PBS or with tunicamycin (1 mg kg^−1^ body weight). At 5 days post injection (dpi) peritoneal exudate was isolated and analyzed for % infected peritoneal exudate cells (PECs) (**B**) intracellular *Toxoplasma* burden in isolated PECs, and (**C**) extracellular parasites (n=5). (**D**-**F**) As for (A-C) but at 7 dpi.
